# Enhanced valorization of Korshinsk peashrub (*Caragana korshinskii* Kom.) as ruminant feed via the selective ligninolysis by the white rot fungus *Dichomitus squalens*

**DOI:** 10.1186/s40104-026-01404-z

**Published:** 2026-05-12

**Authors:** Jiale Liao, Wenjing Zhang, Yidi Wang, Bin Yao, Le Gao, Chuncheng Xu, Haitao Yu, Xing Qin, Guijie Zhang

**Affiliations:** 1 College of Forestry and Prataculture, Ningxia University, Yinchuan, 750021 PR China; 2 College of Animal Science and Technology, Ningxia University, Yinchuan, 750021 PR China; 3 State Key Laboratory of Animal Nutrition and Feeding, Institute of Animal Science, Chinese Academy of Agricultural Sciences, Beijing, 10093 PR China; 4 Tianjin Institute of Industrial Biotechnology, Chinese Academy of Sciences, No. 32, Xiqi Road, Tianjin Airport Economic Park, Tianjin, 300308 PR China; 5 College of Engineering, China Agricultural University, Beijing, 100083 PR China; 6 State Key Laboratory of Animal Nutrition and Feeding, College of Animal Science and Technology, China Agricultural University, Beijing, 100193 PR China

**Keywords:** Korshinsk peashrub, Ligninolytic enzymes, Ruminant feed, Selective biodegradation, White rot fungi

## Abstract

**Background:**

Korshinsk peashrub (*Caragana korshinskii* Kom.) is a valuable forage shrub for ruminants due to its abundant cell walls. However, its utilization is largely limited by the cross-linked structures of lignocellulose within its cell walls. White rot fungi possess the ability to degrade these resistant cross-linked structures, offering enormous potential to develop cost-effective biopretreatment processes of Korshinsk peashrub.

**Results:**

Among the white rot fungi evaluated, *Dichomitus squalens* demonstrated superior efficacy in improving lignocellulose deconstruction and subsequent rumen fermentation of Korshinsk peashrub (*P* < 0.05). This fungus preferentially degrades lignin, hemicellulose, and pectin (*P* < 0.05), which corresponded to significantly improved enzymatic saccharification, ruminal degradability, and gas production (*P* < 0.05). Genomic analysis revealed that *D. squalens* possesses a comprehensive range of genes encoding ligninolytic enzyme. Elevated activities and expression levels of laccase, manganese peroxidase, esterase, glutathione *S*-transferase, versatile peroxidase, and hydrogen peroxide-generating enzymes aligned with the disruption of cross-linked structures and increased porosity of Korshinsk peashrub. Furthermore, the extracellular enzyme cocktail from *D. squalens* exhibited robust lignin‑degrading capability, corroborating its role in selective ligninolysis.

**Conclusions:**

Pretreatment of Korshinsk peashrub with selective white rot fungi offers a practical approach to valorize this woody biomass as an alternative feedstock for ruminants.

**Supplementary Information:**

The online version contains supplementary material available at 10.1186/s40104-026-01404-z.

## Background

Woody plants are a primary source of lignocellulosic biomass, and the carbohydrates released from this biomass can serve as a vital energy supplement for ruminants. The genus *Caragana* (leguminous shrubs) comprises over 100 species, and is widely distributed across East Asia, Eastern Europe, and North America [1]. Representative species included *Caragana arborescens* Lam., *Caragana kozlowii* Kom., *Caragana korshinskii* Kom., *Caragana* *microphylla* Lam., *Caragana jubata *(Pall.) Poir., and *Caragana turkestanica* Kom Korshinsk peashrub (*C. korshinskii* Kom.) holds significant forage and ecological value, and has been extensively cultivated across approximately 8 million hectares in the semi-arid and arid regions of Northwest China [2]. It is harvested once every 3 to 5 years with a stubble height of 10 to 15 cm to rejuvenate growth vigor and prevent aging, generating 40 million tons of Korshinsk peashrub biomass annually in China [3]. Despite structural carbohydrates (cellulose and hemicellulose) accounting for over 50% of its dry matter, the high content of lignin (approximately 16.0%) in Korshinsk peashrub significantly impedes its utilization [4]. The three-dimensional polymer network of lignin and its covalent linkages with hemicellulose together form a recalcitrant, cross-linked structures of lignocellulose, which consequently impedes the hydrolysis of carbohydrates in lignocellulose by glycoside hydrolases from rumen microorganisms [5, 6]. It is worth noting that the lignin can be effectively decomposed by certain fungi and bacteria under aerobic conditions, whereas its decomposition is typically very limited within the anaerobic rumen environment [7]. Therefore, developing an effective pretreatment strategy for Korshinsk peashrub is crucial to realize its value as ruminant feed. An ideal pretreatment should maximize fermentable sugar yields by achieving effective lignin deconstruction while minimizing carbohydrate loss, thereby enhancing cellulose accessibility for enzymatic hydrolysis.

Lignin, a complex polymer, is formed through the oxidative coupling of guaiacyl (G), syringyl (S), and *p*-coumaryl (H) monomers, which are linked together via β-5, β-β and β-O-4 linkages [8]. The diversity of covalent linkages in lignin determines its structural complexity and recalcitrance. Currently, physical and chemical pretreatment methods, such as steam explosion, acid, and alkali, have been widely used in the conversion of lignin in biomass [9]. However, these pretreatment strategies often come with environmental and economic concerns, such as toxic chemical pollution, more energy requirements, high fixed costs and low recovery. White rot fungi have been proposed as a more environmentally friendly and cost-effective alternative to traditional physical and chemical pre-treatment methods for lignin deconstruction in biomass [10]. Lignin degradation is dominated by the peroxidase and laccase secreted by white rot fungi, and is also assisted by free radicals generated via its Fenton reaction, which are then mineralized into the CO_2_ and H_2_O through the central carbon metabolism [11]. Besides the well-known lignin oxidases and auxiliary enzymes, white rot fungi can secrete a broad array of carbohydrate esterases, such as 4-O-methyl-glucuronoyl methylesterase, and then break down the ester bonds connecting glucuronoxylan to lignin [12]. Consequently, the removal of lignin and its linkages with carbohydrates is anticipated to enhance enzymatic contact efficiency and subsequent rumen fermentation.

White rot fungi are commonly categorized into two principal types, "selective" and "simultaneous" rot fungi, based on their decomposition modes for plant cell wall components [13]. In selective rot, lignin and hemicellulose are preferentially degraded, consequently cellulose is selectively retained. In contrast, simultaneous rot fungi lead to a uniform depletion of lignin, cellulose and hemicellulose. Notably, whether white rot fungi deployed either a selective or simultaneous rot strategy mainly depends on the fungal responses to the substrate structure, constituent components and fermentation method during dacay [13]. White rot fungi *Dichomitus squalens*, *Bjerkandera* sp., *Ceriporiopsis subvermispora* and *Phanerochaete chrysosporium* have been reported to colonize wood and straw biomass in nature [14, 15], but it remains unknown whether these four fungi have the selectivity for lignin, cellulose and hemicellulose in Korshinsk peashrub. Hence, this study aims to elucidate whether four fungi cause selective or simultaneous rot or both in Korshinsk peashrub, and to evaluate their biodegradation efficacy and feed efficiency by analyzing changes in chemical composition, enzymatic saccharification, rumen fermentation products, in vitro rumen digestibility and gas production. Integrated analysis of genomics, proteomics, and structural characterization was further employed to elucidate the role of fungal extracellular enzymes in the deconstruction of Korshinsk peashrub.

## Methods

### Fungi seed preparation and their fermentation process on Korshinsk peashrub

The Korshinsk peashrub was harvested from Wuzhong in China (N37°25′, E106°03′, elevation 1,339 m) in 2023. *D. squalens*, *Bjerkandera* sp., *C. subvermispora* and *P. chrysosporium* were purchased from the China General Microbiological Culture Collection Center. The potato dextrose agar plates containing fungal mycelium were preserved at 4 °C. A fungal agar square from the 4 °C stock was aseptically transferred to the center of a fresh potato dextrose agar plate and incubated at 30 °C and 85% relative humidity for 7 d. Then, the resulting mycelium-covered potato dextrose agar medium was mixed with 100 mL of sterile water in a JJ-2 homogenizer (Fangke Instrument Co. Ltd., Jiangsu, China) and homogenized for 60 s at speed 20 r/min. The resulting fungal suspension was inoculated into potato dextrose broth and cultured at 30 °C and 150 r/min for 7 d to obtain a fungal seed culture. The 3.0 g of dried raw material were ground to a 60–80 mesh powder and mixed with 7.5 mL of sterile water in a 250-mL flask. Each flask was then sealed with a high water-resistance plastic membrane. Finally, 5 mL of the seed culture was inoculated into each flask and then cultured at 30 °C with 70.0% relative humidity for 14, 21, and 28 d.

### Chemical composition analysis

Cellulose, hemicellulose, and lignin contents in Korshinsk peashrub were quantified according to a standard analytical procedure from the National Renewable Energy Laboratory (Golden, Colorado, USA). Pectin in Korshinsk peashrub was extracted using an oxalic acid-based extraction method [16]. According to the China National Standard GB/T 20806–2022 [17], neutral detergent fiber (NDF) was measured using the F57 fiber bag technique (ANKOM Technology, Fairport, NY, USA). Neutral detergent solubles (NDS) was calculated as the difference in weight between dry matter and NDF. The concentration of water-soluble carbohydrates (WSC) was measured by the anthrone-concentrated sulphuric acid method [18] using a UV–visible spectrophotometer. All measurements were performed with three independent biological replicates, and three technical replicates were set for each biological replicate.

### Protein extraction and enzyme activity assays

Fungal cultures at various time points were mixed with sterile water at a ratio of 1:4 (g/mL). The mixture was extracted for 2 h at 4 °C and 200 r/min. The filtrates were collected by centrifugation at 12,000 × *g* for 20 min at 4 °C and were then subjected to enzyme activity assay. Total cellulase, exoglucanase and xylanase activities were assayed by the 3,5-dinitrosalicylic acid (DNS) colorimetric method [19]. The total cellulase activity was assayed using Whatman No. 1 filter paper as the substrate in a 200 µL reaction mixture containing 180 µL of 50 mmol/L sodium acetate buffer (pH 4.8) and 20 µL of diluted enzyme extract. The mixture was incubated at 50 °C for 30 min, followed by absorbance measurement at 540 nm. Exoglucanase activity was assayed using 0.5% carboxymethyl cellulose as the substrate in 50 mmol/L sodium acetate buffer (pH 5.0) with 20 μL of diluted enzyme extract. After incubation at 50 °C for 30 min, the absorbance was recorded at 540 nm. Xylanase activity was assayed using 1.0% beechwood xylan as the substrate in 50 mmol/L disodium hydrogen phosphate-citric acid buffer (pH 5.0) with 10 μL of diluted enzyme extract. After incubation at 50 °C for 10 min, the absorbance was recorded at 540 nm.

The activities of β-glucosidase, laccase, manganese peroxidase, total peroxidase, cellobiose dehydrogenase, and esterase were determined according to a previously established method with slight modifications [19]. β-Glucosidase activity was assayed using 4-nitrophenyl-β-D-glucopyranoside as the substrate. A 200-μL reaction mixture containing 190 μL of 4-nitrophenyl-β-D-glucopyranoside (1 mmol/L) and 10 μL of diluted enzyme extract was incubated at 50 °C for 10 min, and the absorbance was read at 405 nm. Laccase activity was assayed using 2,2-azino-bis-3-ethylthiazoline-6-sulfonate (ABTS, 1 mmol/L) as the substrate in 50 mmol/L sodium acetate buffer (pH 4.8) with 20 μL of diluted enzyme extract and 110 μL of ddH_2_O. The absorbance was recorded for 3 min at 420 nm. Manganese peroxidase (MnP) activity was assayed using MnSO_4_ (1 mmol/L) as substrate in 80 mmol/L sodium malonate buffer (pH 4.5) with 20 μL diluted enzyme extract. The H_2_O_2_ (0.1 mmol/L) was used to initiate the reaction, and the absorbance was read for 3 min at 405 nm. Total peroxidase activity was evaluated using ABTS and MnSO_4_ as substrates and the absorbance was read for 3 min at 420 nm. The glutathione *S*-transferase (GST) activity was measured with a colorimetric GST assay kit (Nanjing Jiancheng Bioengineering Institute, Jiangsu, China) using colorimetric method. The cellobiose dehydrogenase (CDH) activity was measured in a reaction system containing 10 μL of diluted enzyme extract and 20 μL of lactose (30 mmol/L) in 50 mmol/L sodium acetate buffer (pH 4.8). This reaction was initiated by 20 μL of 2,6-dichlorophenol indophenol (3 mmol/L) and the absorbance was read for 3 min at 520 nm. Esterase activity was assayed using *p*-nitrophenyl butyrate as the substrate, and the absorbance was read for 3 min at 348 nm. Pectinase activity was determined by the DNS colorimetric method, monitoring absorbance at 540 nm with a UV-visible spectrophotometer [20]. All measurements were conducted with three independent biological replicates, each of which included three technical replicates.

### Measurement of Fe^3+^ reduction capacity, H_2_O_2_ and ·OH levels in the fungal supernatants

The Fe^3+^ reduction capacity of the fungal supernatants was measured based on a stable magenta-colored complex formed by the reaction between ferrozine [3-(2-pyridyl)-5,6-bis(4-phenylsulfonic acid)-1,2,4-triazine] and divalent iron, then determined the absorbance values at 562 nm [21]. The H_2_O_2_ concentration in the fungal supernatants was determined based on the high fluorescence halogen formed by the reaction of Amplex™ Ultra Red probe (Aladdin, Shanghai, China) and H_2_O_2_ under horseradish peroxidase (Aladdin, Shanghai, China) action [22]. The fluorescence intensity was monitored at the excitation and emission wavelengths of 560 nm and 590 nm. According to the previously established method [23], the ·OH level in the fungal supernatants was estimated by monitoring thiobarbituric acid reactive substances (TBARS) at the UV absorption wavelength of 532 nm. All measurements were conducted with three independent biological replicates, each of which included three technical replicates.

### Measurement of enzymatic saccharification efficiency and in vitro rumen digestibility

Enzymatic hydrolysis was performed in a 600-μL reaction system containing cellulase (30 FPU/g substrate; SIGMA-ALDRICH, USA), 50 μg/mL tetracycline, and 50 mmol/L acetic acid-sodium buffer (pH 5.0) with a 3.0% substrate consistency. After enzymatic hydrolysis for 72 h at 30 °C and 150 r/min, the hydrolyzed substrates were centrifuged at 8,000 × *g* and 4 °C for 5 min, and the supernatant was collected. The reducing sugar yield (RSY) was determined according to the DNS colorimetric method at 540 nm using a microplate reader (Synergy HT, BioTek Instruments, USA). The enzymatic RSY was used to evaluate enzymatic saccharification efficiency.

The in vitro rumen digestibility and gas production were performed as previously described [24] using the Daisy II incubator (ANKOM Technology, Fairport, NY, USA). Six healthy male Tan lambs (Tianyuan Well-Bred Lambs Breeding Co., Ltd., Wuzhong, China) with a similar weight and an average age of 9 months were randomly selected to serve as donors of rumen fluid, and their dietary formulas are shown in Table S1 (Additional file 1). The in vitro dry matter digestibility (IVDMD) was calculated as the difference between residues before and after digestion. The in vitro rumen gas production (IVGP) over 48 h was recorded in real time by a pressure sensor (Fortuna, Haberle Labortechnik, Germany) in each fermentation bottle. The rumen pH was determined using a hand-held pH meter (Testo 205, Germany). The NH_3_-N concentration was quantified by the phenol sodium hypochlorite colorimetric method [25]. The concentrations of acetate, propionate, valerate, butyrate, isovalerate, and isobutyrate in rumen were measured using gas chromatography (GC-2000A, Shimadzu Technologies Inc., Japan) following our previously established method [26].

### X-ray diffraction (XRD), Fourier transform infrared spectroscopy (FT-IR) and scanning electron micrograph analysis

The changes in the functional group of wood and alkali lignin samples were measured through FT-IR spectra. Prior to the spectrum measurement, 60-mesh sample powders were uniformly mixed with KBr at a weight ratio of 1:100 and then pressed into thin sheets using the FW-4A tablet press (Nuolei Xinda Technology Co., Ltd., Tianjin, China). The FT-IR spectrum of all samples in the range of 4000–400 cm^−1^ was collected in the absorbance mode. Each sample is scanned 64 times at a resolution of 4 cm^−1^. The crystallinity of the cellulose in the Korshinsk peashrub was determined using an UItima IV XRD instrument. The working conditions were set as follows: decluttering potential, 40 kV and 20 mA; 2Theta (2θ), 5–90^◦^; scanning rate, 8^◦^/min. The crystallinity index was calculated by the peak intensity method. The microscopic structure was observed using a scanning electron micrograph (TM3000, Hitachi, Tokyo, Japan) at an accelerating voltage of 10 kV and a magnification of 1.2 K.

### Evaluation of cellulose bioaccessibility

Based on a modified Simons' staining method [27], the cellulose bioaccessibility of Korshinsk peashrub before and after fungal pretreatment was evaluated using two dyes, direct orange 15 (DO15, 5–36 nm) and direct blue 1 (DB1, ~ 1 nm). Dye concentrations in the supernatant are determined by monitoring the absorbance changes of DB1 at 624 nm, and DO15 at 455 nm using a spectrophotometer. Cellulase (SIGMA-ALDRICH, USA) adsorption capacity was evaluated in a reaction system containing 100 mg Korshinsk peashrub and 10 mL cellulase solution with varying concentrations (0.25, 0.75, 1.0, 1.5, 2.0, 3.0 mg/mL) in 50 mmol/L sodium acetate buffer (pH 4.8). Absorbance at 562 nm was measured to quantify the free cellulase concentration in the supernatant using a bicinchoninic acid protein assay. Adsorption capacities for the dyes and cellulase were calculated as the difference in dye/enzyme concentration and those in the final supernatant. The maximum adsorption capacities of DO15, DB1, and cellulase were calculated by fitting the Langmuir adsorption isothermal model. All measurements were conducted with three independent biological replicates, each of which included three technical replicates.

### Analysis of surface area and pore size distribution

According to an established method [28], the specific surface area and pore size distribution of all samples were characterized from N_2_ adsorption–desorption isotherms collected at 77 K using a volumetric adsorption apparatus (ASAP 2460, Micromeritics, USA). Prior to analysis, each sample was degassed at 120 °C for 10 h. The Brunauer–Emmett–Teller specific surface area was calculated based on the N_2_ adsorption data in the relative pressure range of 0.005 to 0.300 at 77 K. The pore size distribution was determined from the adsorption branch of the N_2_ isotherms using Non-Local Density Functional Theory with a slit-pore model assumption. At a relative pressure of 0.99, the micropore area and volume were estimated from the N_2_ adsorption amount using the t-plot method.

### Statistical analysis

All data were showed as means ± standard error of the mean (SEM). The trial employed a 4 × 4 factorial design with two fixed variables: white rot fungi and incubation time. The chemical composition, enzymatic saccharification efficiency and in vitro rumen fermentation data were analyzed using a linear mixed multivariable model in IBM-SPSS version 26.0 (SPSS Inc., Chicago, IL, USA):

$${y}_{ij} = \mu +{W}_{i}+{T}_{j}+{\left(W\times T\right)}_{ij}+{e}_{ij}$$

Where *y*_*ij*_ represents the response variable (NDF, NDS, WSC, cellulose, hemicellulose, lignin, pectin, RSY, IVDMD and IVGP) and *μ* is the general mean; *W*_*i*_ represents the fixed effect of white rot fungi (*i* = 1, 2, 3, 4); *T*_*j*_ represents the fixed effect of incubation time (*j* = 1, 2, 3, 4); (*W* × *T*)_*ij*_ represents the fixed interaction effect between white rot fungi and incubation time;* e*_*ij*_ is the residual error. Tukey's multiple comparison was conducted to compare the differences among treatments, and a declared *P* value < 0.05 represented a statistical significance. The infrared transmittance was transformed into absorbance with the baseline correction and normalization by Omnic 32 (Nicolet, New York, USA).

## Results

### Comparative analysis of fungal pretreatment efficacy based on chemical composition, enzymatic saccharification and in vitro rumen fermentation

The changes in chemical composition of Korshinsk peashrub after fungal pretreatments are presented in Table 1 and Table S2 (Additional file 1). The NDF content of Korshinsk peashrub in all fungal treatments significantly decreased with time, while the NDS content significantly increased (*P* < 0.05). It is worth noting that *D. squalens* demonstrated the most obvious influence on NDF and NDS, followed by *Bjerkandera* sp. Although a significant decrease in WSC was observed in all treatment groups (*P* < 0.01), the declining trend in *D. squalens* treatment was least. *D. squalens* had minimal cellulose loss among all fungi. After treatments with *D. squalens*, *Bjerkandera* sp. and *C. subvermispora*, lignin and hemicellulose contents significantly decreased over time (*P* < 0.01). Comparatively, *D. squalens* demonstrated the strongest biodegradation capability. A net loss in pectin was only observed in *D. squalens* treatment. The interaction between white rot fungi and incubation time (W × T) was statistically significant for NDF, NDS, WSC, cellulose, hemicellulose, lignin, and pectin (*P* < 0.001).

**Table 1:** Chemical compositions analysis of Korshinsk peashrub decomposed by different white rot fungi (*n* = 3)

Items	White rot fungi (*W*)	CON	Incubation time (*T*)			SEM	*P* value		
			14 d	21 d	28 d		*W*	*T*	*W* × *T*
NDF, %DW	*D. squalens*	70.36^A,a^	66.01^C,b^	63.39^D,c^	61.13^D,d^	0.055	< 0.001	< 0.001	< 0.001
	*Bjerkandera* sp.	70.81^A,a^	67.98^B,b^	66.88^C,c^	66.26^C,c^				
	*C. subvermispora*	70.48^A,a^	68.31^B,b^	67.94^B,b^	66.81^B,c^				
	*P. chrysosporium*	70.19^A,a^	69.48^A,b^	68.43^A,c^	67.60^A,d^				
NDS, %DW	*D. squalens*	29.64^A,d^	33.99^A,c^	36.61^A,b^	38.87^A,a^	0.055	< 0.001	< 0.001	< 0.001
	*Bjerkandera* sp.	29.19^A,c^	32.02^B,b^	33.12^B,a^	33.74^B,a^				
	*C. subvermispora*	29.52^A,c^	31.69^B,b^	32.06^C,b^	33.19^C,a^				
	*P. chrysosporium*	29.81^A,d^	30.52^C,c^	31.58^D,b^	32.40^D,a^				
WSC, %DW	*D. squalens*	37.52^A,a^	35.54^BC,b^	34.56^A,bc^	33.72^A,c^	0.075	< 0.001	< 0.001	< 0.001
	*Bjerkandera* sp.	37.91^A,a^	34.56^C,b^	33.67^B,bc^	32.78^B,c^				
	*C. subvermispora*	38.09^A,a^	35.96^B,b^	33.08^B,c^	30.62^C,d^				
	*P. chrysosporium*	37.97^A,a^	38.03^A,a^	31.39^C,b^	28.47^D,c^				
Cellulose, %DW	*D. squalens*	39.00^A,a^	39.10^A,a^	38.83^A,ab^	38.58^A,b^	0.031	< 0.001	< 0.001	< 0.001
	*Bjerkandera* sp.	39.29^A,a^	38.60^BC,b^	38.18^AB,c^	37.98^B,c^				
	*C. subvermispora*	38.94^A,a^	38.41^C,b^	37.46^B,c^	37.70^C,c^				
	*P. chrysosporium*	39.14^A,a^	38.67^B,a^	37.89^B,b^	37.48^C,b^				
Hemicellulose, %DW	*D. squalens*	16.44^A,a^	14.24^B,b^	12.75^C,c^	11.72^B,d^	0.039	< 0.001	< 0.001	< 0.001
	*Bjerkandera* sp.	16.57^A,a^	15.79^A,b^	15.70^B,b^	15.64^A,b^				
	*C. subvermispora*	16.29^A,a^	15.81^A,b^	16.69^A,a^	15.87^A,b^				
	*P. chrysosporium*	16.36^A,a^	16.00^A,b^	16.07^B,b^	15.91^A,b^				
Lignin, %DW	*D. squalens*	14.91^A,a^	12.68^D,b^	11.82^D,c^	10.83^D,d^	0.024	< 0.001	< 0.001	< 0.001
	*Bjerkandera* sp.	15.02^A,a^	13.60^C,b^	12.99^C,c^	12.63^C,d^				
	*C. subvermispora*	14.83^A,a^	14.09^B,b^	13.78^B,c^	13.24^B,d^				
	*P. chrysosporium*	14.86^A,a^	14.81^A,a^	14.47^A,ab^	14.28^A,b^				
Pectin, %DW	*D. squalens*	3.63^A,a^	2.77^B,b^	1.85^C,c^	1.38^C,d^	0.009	< 0.001	< 0.001	< 0.001
	*Bjerkandera* sp.	3.66^A,a^	3.59^A,ab^	3.41^B,c^	3.44^B,bc^				
	*C. subvermispora*	3.71^A,a^	3.57^A,b^	3.47^B,c^	3.42^B,c^				
	*P. chrysosporium*	3.57^A,a^	3.66^A,a^	3.69^A,a^	3.59^A,a^				

*NDF* Neutral detergent fiber, *NDS* Neutral detergent solubles, *WSC* Water soluble carbohydrates, *DW* Dry weight, *CON* Raw materials with no fungi treatment, *SEM* Standard error of mean

*W* represents the fixed effect of white rot fungi; *T* represents the fixed effect of incubation time

^a–d^Mean values with different superscripts in the same row showed a statistical differences at different incubation time (*P* < 0.05)

^A–D^Mean values with different superscripts in the same column showed a statistical difference under different treatments of white rot fungi (*P* < 0.05)

**Fig. 1:**
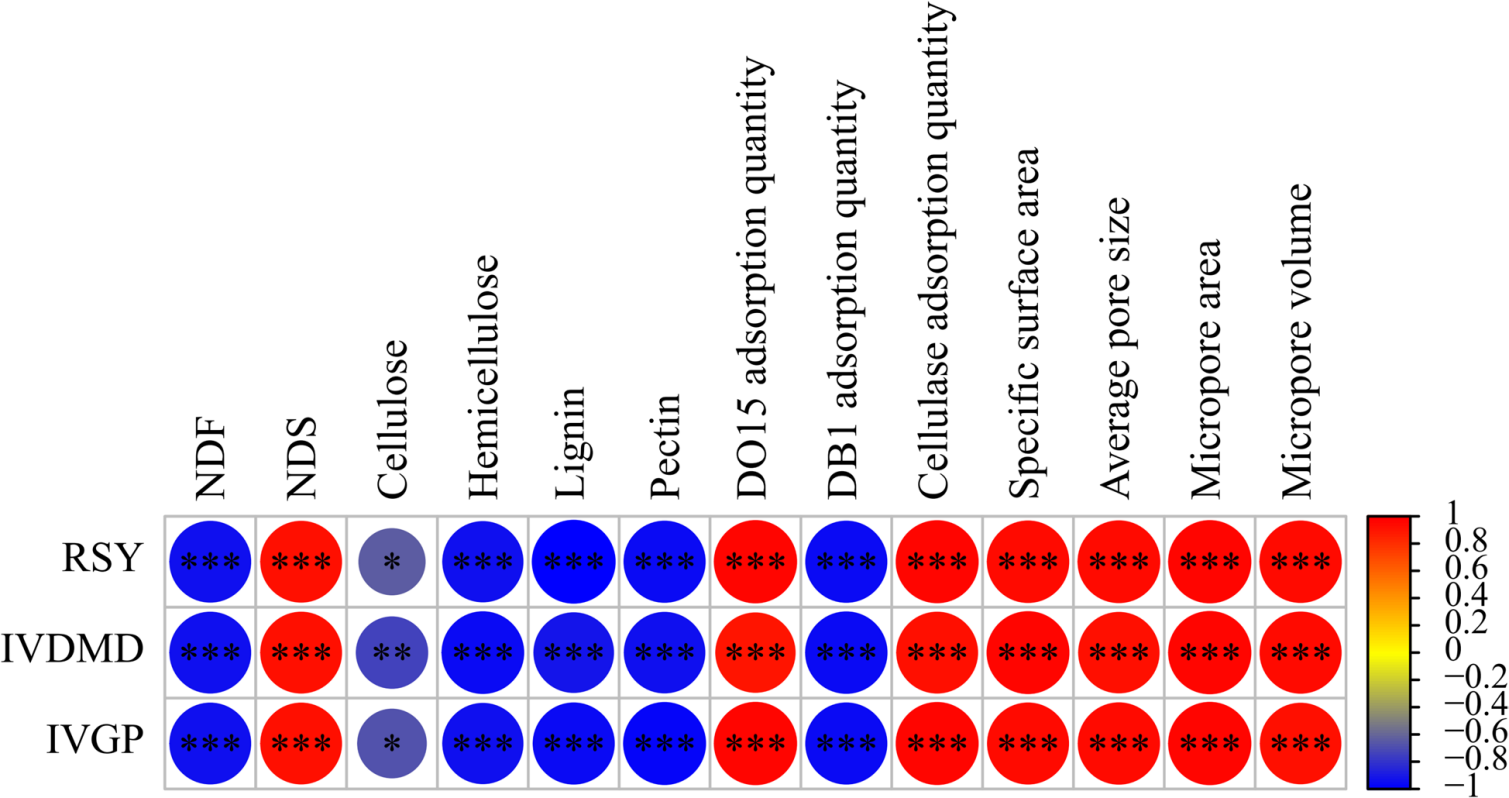
Pearson's correlation analysis among enzymatic saccharification, in vitro rumen fermentation, chemical compositions, cellulose bioaccessibility and pore structure characteristics. A significant correlation is expressed as ^*^P < 0.05, ^**^P < 0.01 and ^***^P < 0.001. Red colors represent positive correlations, whereas blue colors represent negative correlations. *RSY* Reducing sugar yield, *IVDMD* In vitro dry matter digestibility, *IVGP* In vitro gas production, *NDF* Neutral detergent fiber, *NDS* Neutral detergent solubles, *DO15* Direct orange 15, *DB1* Direct blue 1

The results for enzymatic saccharification, in vitro rumen digestibility and gas production are summarized in Table 2. All these fungi caused net increases in the RSY, IVDMD and IVGP (*P* < 0.05), except *P. chrysosporium* which caused a net loss in IVGP. The RSY, IVDMD and IVGP were significantly influenced by the interaction between white rot fungi and incubation time (*W* × *T*; *P* < 0.001). RSY, IVDMD and IVGP of Korshinsk peashrub treated with *D. squalens* were positively correlated with the NDS, specific surface area, average pore size, and adsorption quantities of DO15 and cellulase (*P* < 0.001), while negatively correlated with the NDF, lignin, hemicellulose and pectin (*P* < 0.001; Fig. 1).

**Table 2:** Enzyme saccharification and in vitro rumen degradability of Korshinsk peashrub decomposed by different white rot fungi (*n* = 3)

Items	White rot fungi (*W*)	CON	Incubation time (*T*)			SEM	*P* value		
			14 d	21 d	28 d		*W*	*T*	*W* × *T*
RSY, mg/g DW	*D. squalens*	99.96^A,d^	126.47^A,c^	139.21^A,b^	148.82^A,a^	0.299	< 0.001	< 0.001	< 0.001
	*Bjerkandera* sp.	98.99^A,c^	101.78^B,c^	111.18^B,b^	119.31^B,a^				
	*C. subvermispora*	99.45^A,c^	100.47^B,bc^	104.16^C,b^	108.01^C,a^				
	*P. chrysosporium*	99.14^A,b^	97.33^B,ab^	101.99^C,ab^	102.55^D,a^				
IVDMD, %DW	*D. squalens*	41.32^A,d^	44.12^A,c^	45.75^A,b^	48.44^A,a^	0.073	< 0.001	< 0.001	< 0.001
	*Bjerkandera* sp.	40.63^A,d^	42.62^B,c^	43.57^B,b^	45.25^B,a^				
	*C. subvermispora*	40.73^A,c^	41.01^BC,bc^	41.93^C,b^	43.16^C,a^				
	*P. chrysosporium*	40.96^A,a^	41.85^C,a^	41.73^C,a^	41.88^ D,a^				
IVGP, mL/g OM	*D. squalens*	35.67^B,d^	45.39^A,c^	62.90^A,b^	75.07^A,a^	0.189	< 0.001	< 0.001	< 0.001
	*Bjerkandera* sp.	36.28^B,d^	39.98^B,c^	49.09^B,b^	56.82^B,a^				
	*C. subvermispora*	36.42^B,c^	37.71^C,bc^	38.65^C,ab^	40.34^C,a^				
	*P. chrysosporium*	38.19^A,a^	38.72^C,a^	36.74^C,b^	33.31^D,c^				

*RSY* Reducing sugar yield, *IVDMD* In vitro dry matter digestibility, *IVGP* In vitro gas production, *DW* Dry weight, *OM* Organic matter, *CON* Raw materials with no fungi treatment, *SEM* Standard error of mean

*W* represents the fixed effect of white rot fungi; *T* represents the fixed effect of incubation time

^a–d^Means values with different superscripts in the same row showed a statistical differences at different incubation time (*P* < 0.05)

^A–D^Means values with different superscripts in the same column showed a statistical difference under different treatments of white rot fungi (*P* < 0.05)

Table 3 shows the results of in vitro rumen fermentation products after fungal pretreatments. After 28 days of incubation, Korshinsk peashrub treated with fungi had lower concentrations of NH_3_-N and isovalerate (*P* < 0.001). Korshinsk peashrub treated with *D. squalens* had lower pH value (*P* = 0.021). TVFAs concentration increased significantly after *D. squalens* treatment (*P* < 0.001), whereas a net reduction was observed in *P. chrysosporium* treatment (*P* < 0.001). The isobutyrate concentration and the acetate-to-propionate ratio were significantly reduced by *D. squalens* (*P* < 0.001) and *Bjerkandera* sp. (*P* = 0.001) treatments.

**Table 3:** In vitro rumen fermentation products after pretreatment with white rot fungi for 28 d (*n* = 3)

Items	CON	White rot fungi				SEM	*P* value
		*D. squalens*	*Bjerkandera* sp.	*C. subvermispora*	*P. chrysosporium*		
pH	6.85^ab^	6.76^c^	6.78^bc^	6.80^abc^	6.88^a^	0.014	0.021
NH_3_-N, mg/dL	19.04^a^	14.36^d^	14.96^d^	16.14^c^	17.41^b^	0.463	< 0.001
TVFAs, mmol/L	58.76^b^	61.48^a^	59.72^ab^	58.39^b^	53.89^c^	0.715	< 0.001
Acetate, mmol/L	37.60^ab^	38.60^a^	37.86^ab^	36.92^b^	34.69^c^	0.378	< 0.001
Propionate, mmol/L	13.68^c^	16.08^a^	14.95^b^	14.37^bc^	11.81^d^	0.403	< 0.001
Acetate/propionate	2.75^ab^	2.40^d^	2.53^c^	2.57^bc^	2.95^a^	0.056	0.001
Butyrate, mmol/L	4.10^a^	3.94^a^	3.99^a^	4.05^a^	4.08^a^	0.027	0.350
Valerate, mmol/L	1.01^a^	0.97^a^	1.00^a^	0.95^a^	1.05^a^	0.015	0.254
Isobutyrate, mmol/L	0.93^a^	0.78^c^	0.82^bc^	0.90^ab^	0.96^a^	0.023	0.008
Isovalerate, mmol/L	1.45^a^	1.10^d^	1.18^ cd^	1.21^bc^	1.29^b^	0.034	< 0.001

*TVFAs* Total volatile fatty acids, *CON* Raw materials with no fungi treatment, *SEM* Standard error of mean

^a–d^Means values with different superscripts in the same row showed a statistical differences at different incubation time (*P* < 0.05)

### Genome assembly, function annotation and comparative genomics analysis based on *D. squalens*

**Fig. 2:**
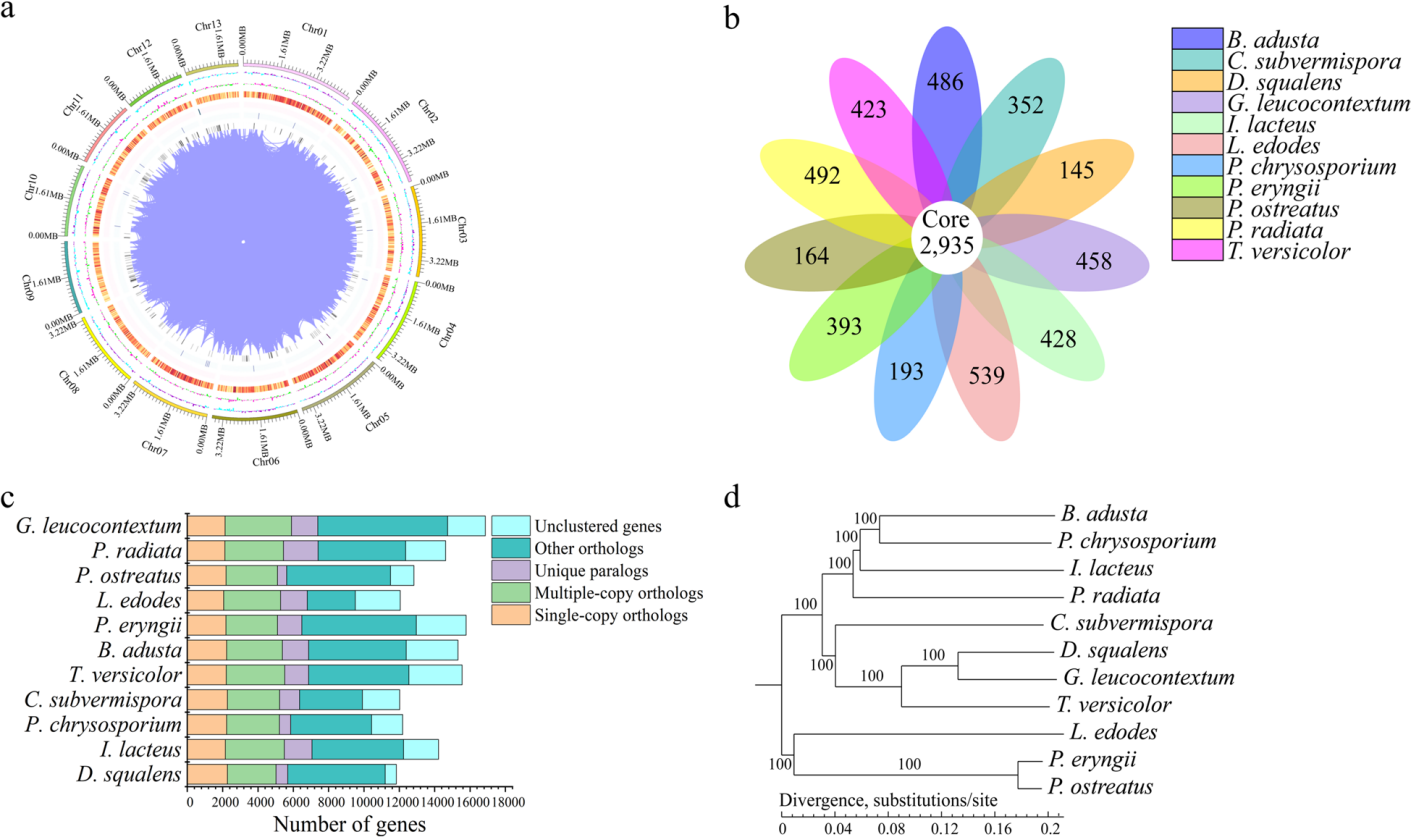
Genome assembly of *D. squalens* and its phylogenomic analysis with other white rot fungi. **a** Whole genome map of *D. squalens*. **b** Number of gene families. **c** Number of orthologous genes. **d** Phylogenomic analysis among different white rot fungi. *Chr* Chromosome

As depicted in Fig. 2a, we assembled a chromosome-level genome map of *D. squalens* with 13 distinct chromosomes. More than 99% of the 11,838 genes and 95% of the total sequences were located on the 13 largest chromosomes (Additional file 1: Table S3 and S4). Each chromosome ranges from approximately 2.0 to 5.0 Mb. The total genomic size of *D. squalens* is 53.78 G, supported by a 1,178.09-fold sequence coverage (Additional file 1: Table S5). All the predicted genes could be precisely annotated to Kyoto Encyclopedia of Genes and Genomes (KEGG) and Gene Ontology (GO) databases (Additional file 1: Table S6). Similar to other lignocellulose-degrading fungi, the largest protein families in *D. squalens* include zinc knuckle, F-box-like, protein kinase domain, major facilitator superfamily, cytochrome P450 monooxygenases and ATP-binding cassette transporter (Additional file 1: Table S7). These families play significant roles in cellular signal transduction, metabolite biosynthesis and transport, and gene expression. For instance, members from major facilitator superfamily and the ATP-binding cassette transporter are indispensable for the transport of sugar and lignin-derived aromatic compounds in *D. squalens*.

A comparative phylogenomic analysis was conducted based on the genomic sequences of *D. squalens* and 10 other sequenced white rot fungi. The results identified more than 2,000 single-copy orthologous genes in the 11 fungi (Fig. 2c). In the phylogenomic analysis, *D. squalens*, *Ganoderma leucocontextum*, *Trametes versicolor* and *C. subvermispora* were located in the adjacent branches with a 100% maximum likelihood bootstrap value in each node (Fig. 2d), indicating a close genetic relationship among them. Distances among the branches showed that *D. squalens* had the closest genetic relationship with *G. leucocontextum*, and a moderate genetic relationship with *T. versicolor* and *C. subvermispora*. These results illustrated that *G. leucocontextum*, *T. versicolor* and *C. subvermispora* may have a similar bioconversion pattern as *D. squalens*.

**Fig. 3:**
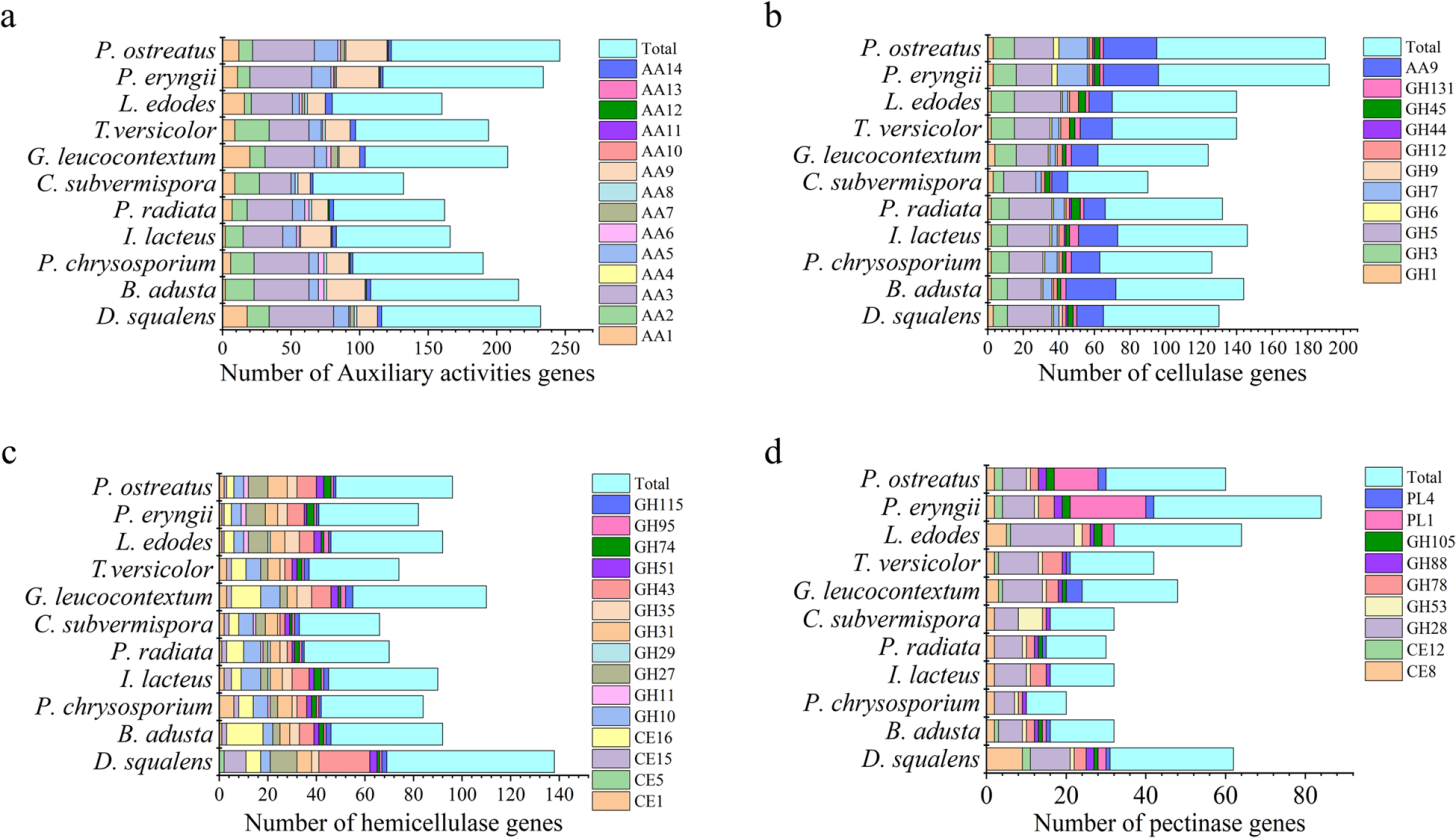
Comparative analysis of genes related to the deconstruction of plant cell wall components. **a** Auxiliary activities genes. **b** Cellulase genes. **c** Hemicellulase genes. **d** Pectinase genes. *AA *Auxiliary activities, *GH *Glycoside hydrolases, *CE *Carbohydrate esterases,* PL *Polysaccharide lyases

Comparative genomics analysis deciphers the genetic codes of *D. squalens* governing the deconstruction of Korshinsk peashrub cell wall. A total of 657 genes in *D. squalens* were annotated into the Carbohydrate-active enzymes (CAZy) database (Additional file 1: Table S8 and S9), including 65 carbohydrate-binding modules, 48 carbohydrate esterases, 274 glycoside hydrolases, 138 glycosyl transferases, 16 polysaccharide lyases and 116 auxiliary activities (AA). Total quantity of these six types of enzymes in *D. squalens* has higher values versus all other tested species. In CAZy (http://www.cazy.org/), laccase is classified into the AA1 family, whereas MnP, versatile peroxidase, lignin peroxidase and dye-decolorizing peroxidase are grouped into the AA2 family. The AA3 and AA5 families included alcohol oxidase, aryl alcohol oxidase, GMC oxidoreductase, CDH and glyoxal oxidase, and their primary function is to produce H_2_O_2_. A comparative genomics analysis shows that hemicellulase genes of *D. squalens* exhibited the largest types and quantity among the 11 sequenced fungi, whereas its cellulase and pectinase genes were comparatively less prevalent. Most importantly, *D. squalens* has also the largest quantity of genes in the AA1, AA2, AA3, and AA5 families (Fig. 3a and additional file 1: Table S9). This result indicates that the genome of *D. squalens* possesses a comprehensive range of genes that encode ligninolytic enzymes, positioning it as one of the most promising lignin degraders in the Basidiomycetes.

### Dynamic changes in lignocellulolytic enzyme activities and secretomics during *D. squalens* colonization

All secreted proteins could be assigned with a definitive function through a homology search against the GO, KEGG, InterPro (IPR) and Eukaryotic Orthologous Groups of proteins (KOG) databases based on the predicted gene set derived from the genome (Additional file 2: Fig. S2). The structure and function of these proteins are primarily involved in oxidation reduction process, hydrogen peroxide production, lignin degradation, and carbohydrate metabolism. For instance, 114 proteins were significantly enriched in IPR terms corresponding to the heme peroxidase domain, GST domain, glucose-methanol-choline oxidoreductase domain, carboxylesterase domain, glycoside hydrolase domain and cellulose-binding domain (Additional file 2: Fig. S2c).

**Fig. 4:**
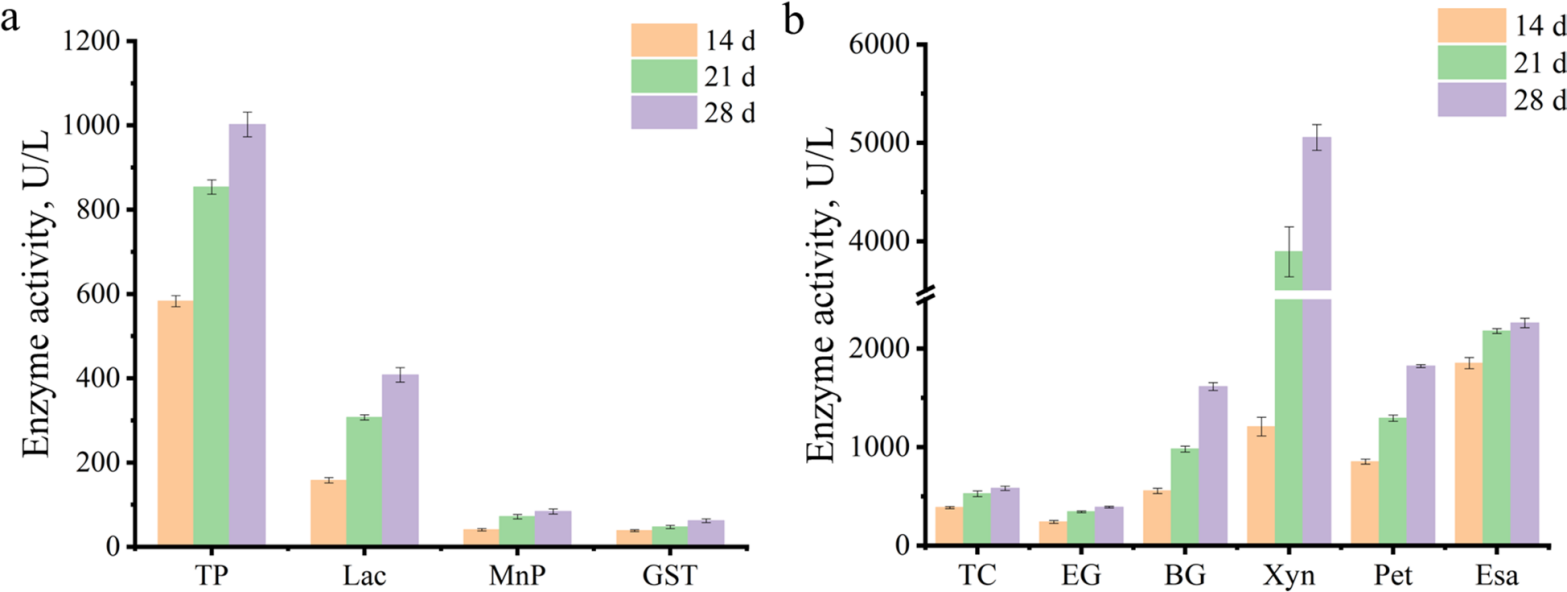
Ligninolytic and carbohydrate enzyme activities in *D. squalens*. **a** Ligninolytic enzyme activities. **b** Carbohydrate enzyme activities. *TP* Total peroxidase, *Lac* Laccase, *MnP* Manganese peroxidase, *GST* Glutathione *S*-transferase, *TC* Total cellulase, *EG* Endonucellase, *BG *β-glucosidase, *Xyn* Xylanase, *Pet* Pectinase, *Esa* Esterase

**Fig. 5:**
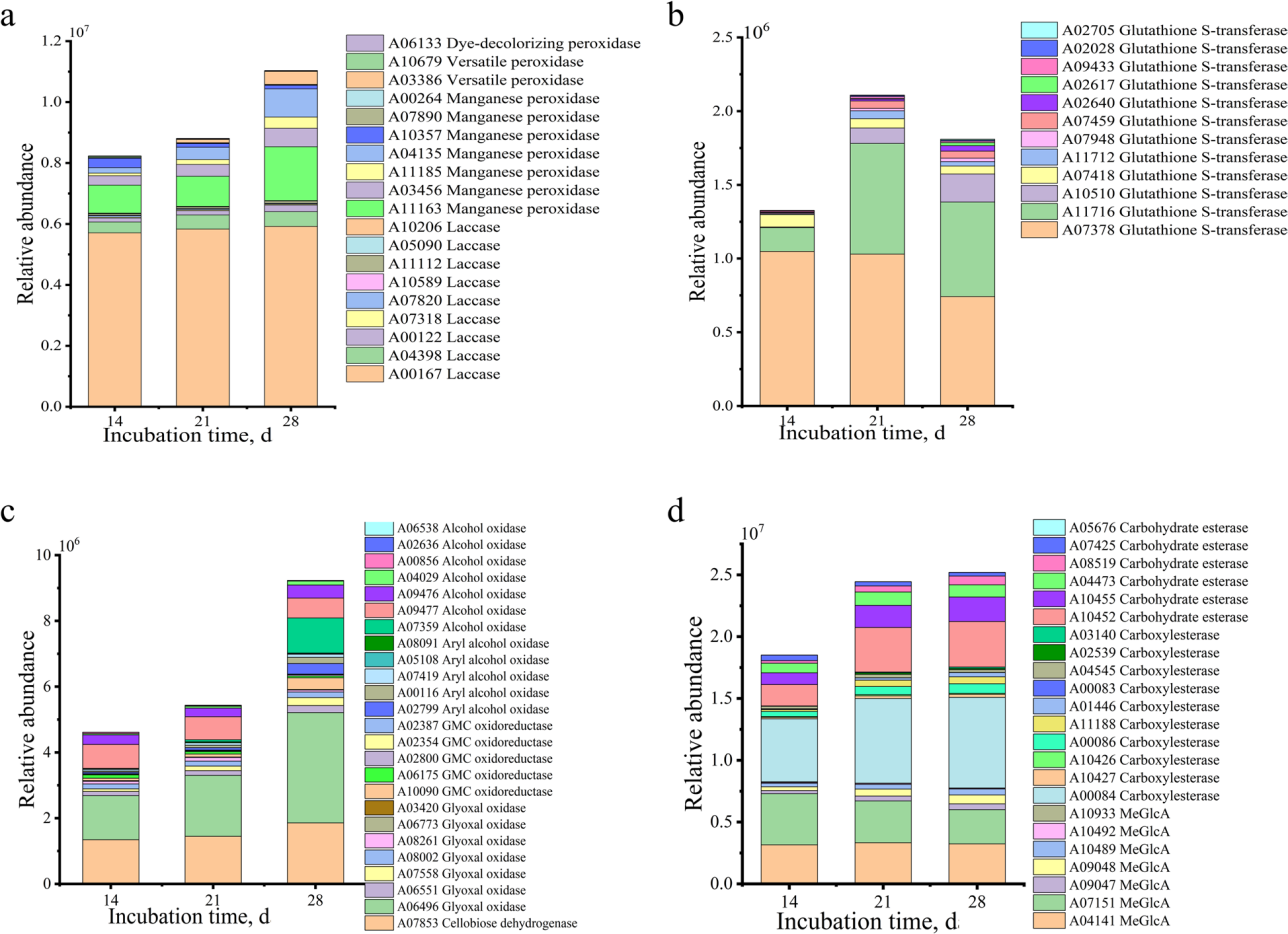
Identification and analysis of extracellular enzymes from *D. squalens* involved in lignin deconstruction. **a** Ligninolytic enzymes. **b** Glutathione *S*-transferase. **c** Hydrogen peroxide-generating enzymes. **d** Carbohydrate esterases. *MeGlcA* 4-O-methyl-glucuronoyl methylesterase

**Fig. 6:**
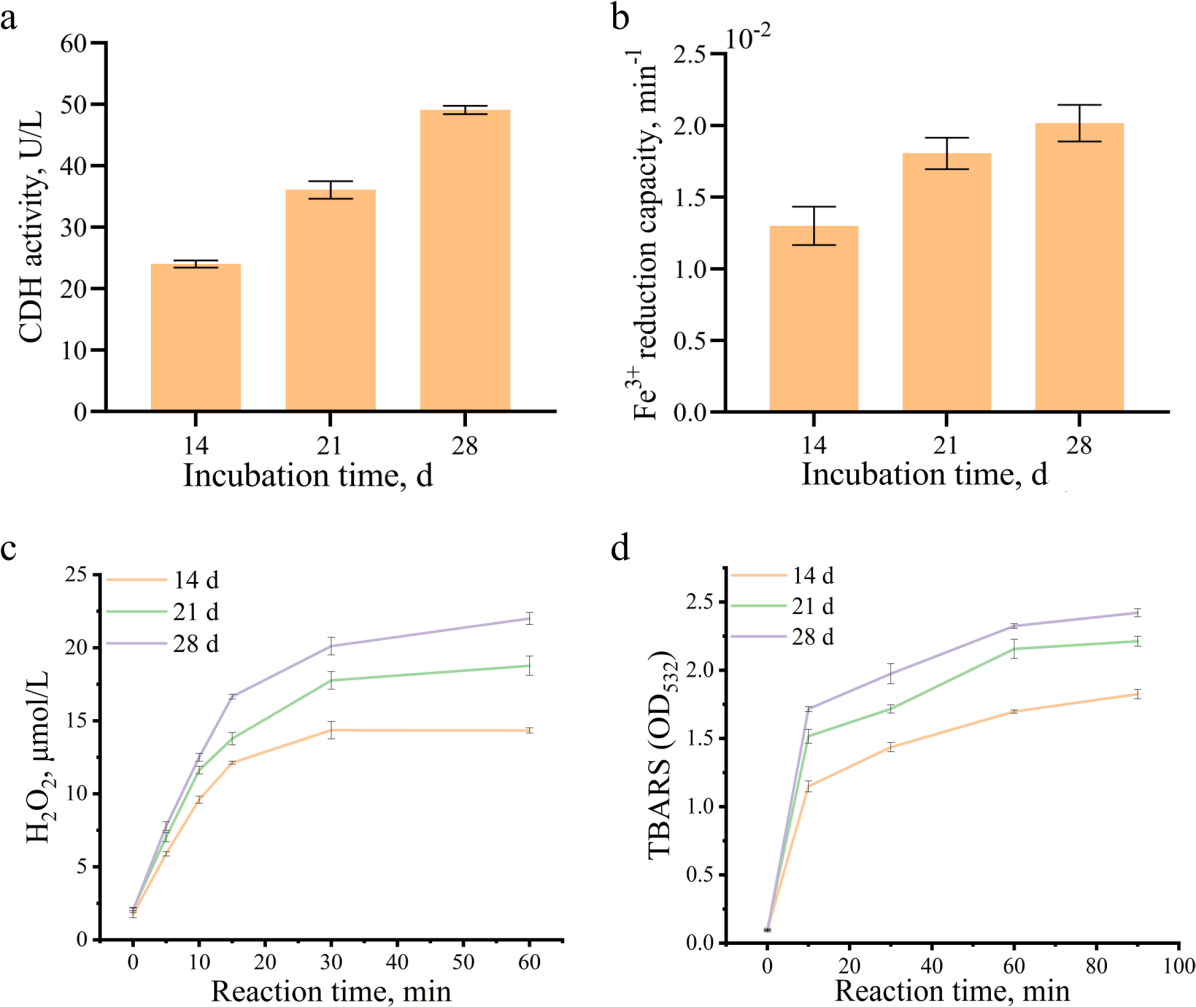
The generation of H_2_O_2_ and ·OH in the presence of cellobiose dehydrogenase. **a** Cellobiose dehydrogenase activity. **b** Fe^3+^ reduction capacity. **c** H_2_O_2_ production. **d** ·OH production. *CDH* Cellobiose dehydrogenase, *TBARS* Thiobarbituric acid reactive substances

Proteomics identified 9 laccase, 7 MnP, 2 versatile peroxidase, 1 dye-decolorizing peroxidase and 12 GST, which together formed a ligninolytic catalytic network (Fig. 4a and b). In *D. squalens*, the activities of ligninolytic enzymes (MnP, laccase, GST, and total peroxidase) exhibited a time-dependent increase, peaking simultaneously at 28 d with respective values of 83.92 U/L*,* 407.88 U/L, 61.77 U/L and 1,002.01 U/L (Fig. 4a). Besides the well-known ligninolytic enzymes (e.g., MnP and laccase), a broad array of auxiliary enzyme-mediated reactions also can modify lignin. We identified 7 glyoxal oxidase, 5 GMC oxidoreductase, 5 aryl alcohol oxidase, 7 alcohol oxidase and 1 CDH, and their primary function is to catalyze the production of H_2_O_2_ (Fig. 5c). Following *D. squalens* intervention, a significant time-dependent increase in H_2_O_2_ and ·OH in extracellular extracts occurred together with a marked enhancement in both Fe^3+^ reduction capacity and CDH activity (Fig. 6; *P* < 0.05).

A total of 78 carbohydrate-degrading enzymes including 34 cellulase, 27 hemicellulase, 11 pectinase and 6 other polysaccharide hydrolases were identified based on proteomics (Additional file 1: Table S10). Activities of cellulase, hemicellulase, and pectinase significantly increased with time, achieving the highest activities of 582.5 U/L for total cellulase, 391.1 U/L for endocellulase, 1,614.6 U/L for β-glucosidase, 5,054.8 U/L for xylanase, and 1,878.6 U/L for pectinase at 28 d (Fig. 4b). The 23 carbohydrate esterases were identified in extracellular enzymes (Fig. 5d), and total esterase activity in *D. squalens* treatment significantly increased over time (*P* < 0.01; Fig. 4b).

### Effects of *D. squalens* pretreatment on the crystallinity, structural characteristics and cellulose bioaccessibility of Korshinsk peashrub

**Fig. 7:**
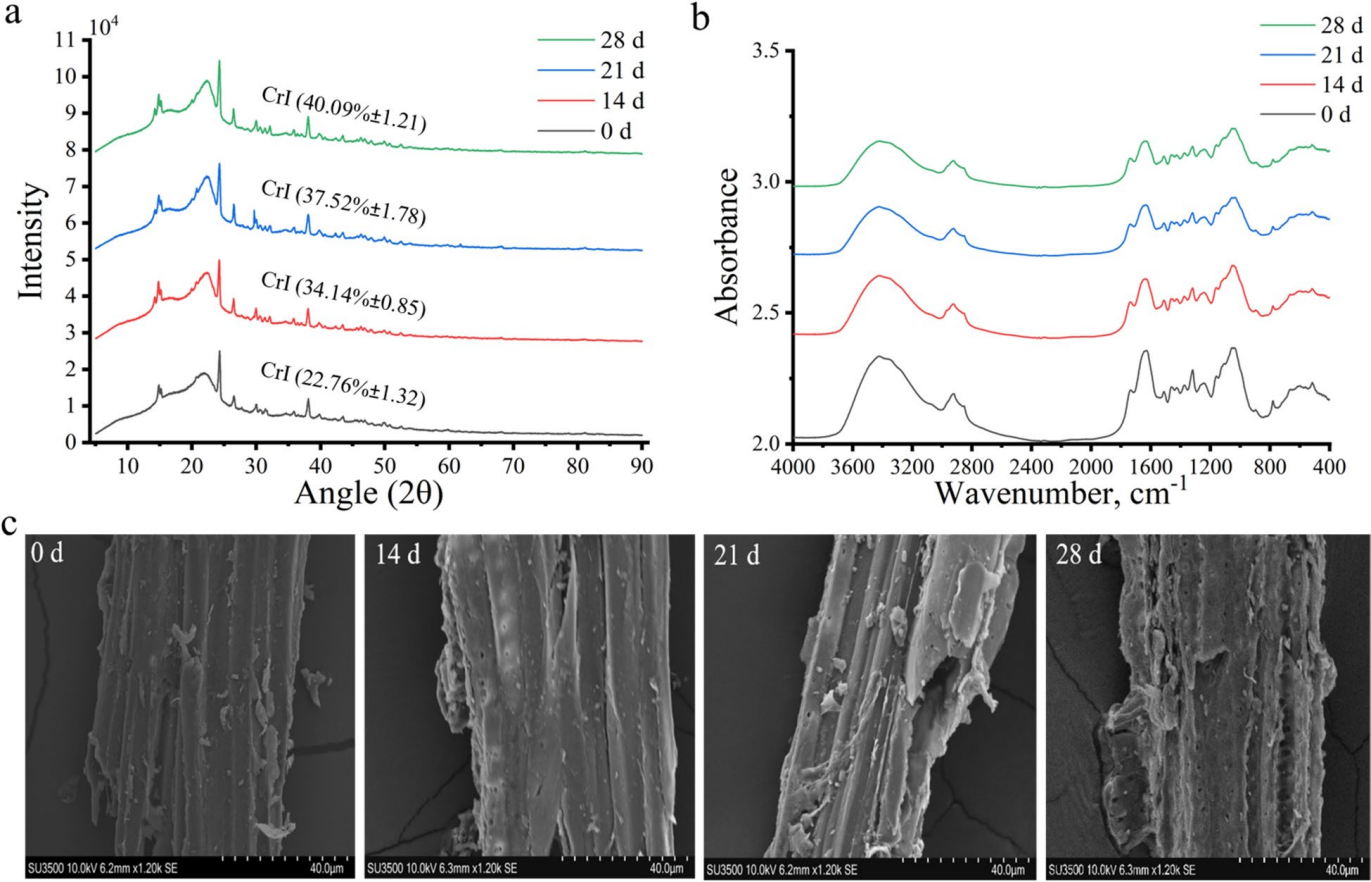
Effects of *D. squalens* treatment on crystalline and structural properties of Korshinsk peashrub. **a** Crystalline characterization. **b** Chemical functional groups. **c** Microstructure observation. *CrI* Crystallinity index

The crystalline characterizations are presented in Fig. 7a. The crystallinity index of Korshinsk peashrub treated by *D. squalens* significantly increased with time (*P* < 0.05). As demonstrated in Fig. 7b and Table S11 (Additional file 1), FT-IR spectra was used to characterize the changes in functional groups of Korshinsk peashrub before and after pretreatment. The bands at 1,107 cm^−1^, 1,242 cm^−1^, 1,319 cm^−1^, 1,425 cm^−1^, 1,510 cm^−1^ and 3,420 cm^−1^ are assigned to functional groups related to the basic units and side chains of lignin (Fig. 7b). These absorption bands quickly shifted to lower wavelengths when subjected to *D. squalens* treatment. There were clear declines at the bands 897 cm^−1^, 1,053 cm^−1^, 1,628 cm^−1^ and 1,736 cm^−1^, corresponding to the functional groups in cellulose, hemicellulose and pectin. Microstructure analysis reveals that the surface of Korshinsk peashrub is dense, smooth, and rigid, whereas Korshinsk peashrub pretreated with fungi exhibited a rough and porous surface (Fig. 7c).

The dye and cellulase adsorption characteristics, and porous structure parameters of Korshinsk peashrub before and after fungal pretreatment are presented in Table 4. The N_2_ adsorption of Korshinsk peashrub begins at the relative pressure range from 0.4 to 0.9, and its N_2_ adsorption capacity reached a saturated state at the relative pressure range from 0.9 to 1.0 (Additional file 2: Fig. S3). The average pore size and specific surface area significantly increased with time (*P* < 0.001; Table 4). The adsorption amounts of DO15 and cellulase significantly increased with time (*P* < 0.001; Table 4), whereas the adsorption amount of DB1 significantly decreased (*P* < 0.001).

**Table 4:** The cellulose bioaccessibility and pore structure characteristics of Korshinsk peashrub decomposed by *D. squalens* (*n* = 3)

Items	CON	Incubation time			SEM	*P* value
		14 d	21 d	28 d		
Dyes adsorption properties						
DO15, mg/g DW	172.09^d^	179.75^c^	186.44^b^	189.50^a^	2.023	< 0.001
DB1, mg/g DW	162.65^a^	157.52^b^	153.25^c^	152.08^d^	1.255	< 0.001
DO15/DB1	1.06^d^	1.14^c^	1.22^b^	1.25^a^	0.022	< 0.001
Cellulase adsorption capacity, mg/g DW	3.36^d^	6.09^c^	8.10^b^	8.93^a^	0.649	< 0.001
Thermophysical properties						
Specific surface area, m^2^/g DW	0.65^d^	0.97^c^	1.02^b^	1.27^a^	0.067	< 0.001
Micropore area, m^2^/g DW	0.06^d^	0.09^c^	0.11^b^	0.12^a^	0.007	< 0.001
Micropore volume, 10^−6^cm^3^/g DW	4.00^d^	7.00^c^	15.00^b^	28.00^a^	0.001	< 0.001
Average pore size, nm	11.34^d^	15.54^c^	16.12^b^	16.94^a^	0.652	< 0.001

*DO15* Direct orange 15, *DB1* Direct blue 1, *DW* dry weight, *SEM* Standard error of mean

^a–d^Means values with different superscripts in the same row indicate significant differences (*P* < 0.05)

### Lignin degradability of extracellular enzyme cocktail (EEC)

**Fig. 8:**
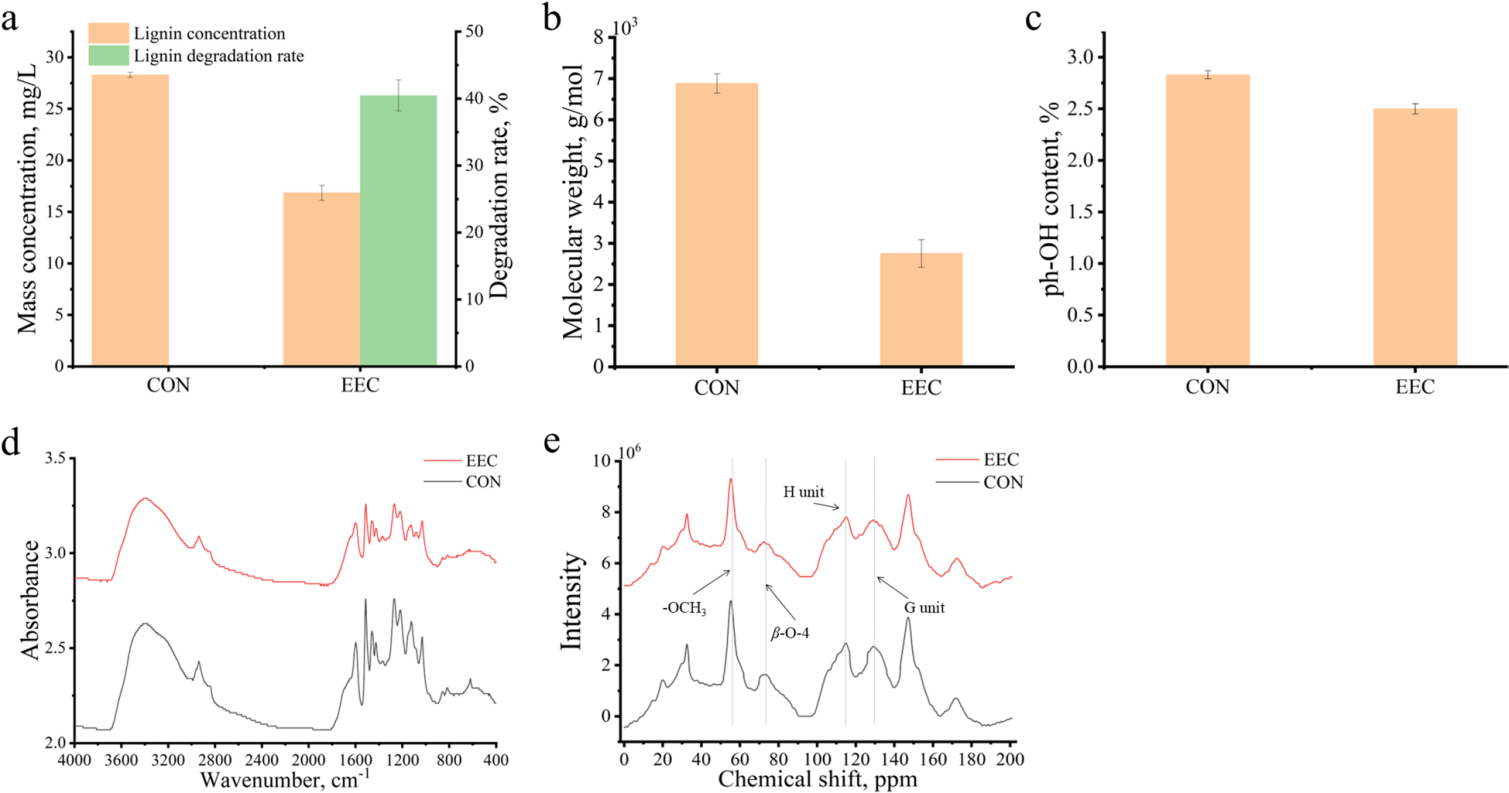
Lignin degradability of extracellular enzyme cocktail from *D. squalens*. **a** Lignin concentration and degradation rate. **b** Molecular weight of lignin. **c** Phenolic hydroxyl content. **d** Fourier transform infrared spectroscopy: Chemical functional groups. **e** Nuclear magnetic resonance ^13^C spectrum: Chemical functional groups. *CON* Control without extracellular enzyme cocktail treatment, *EEC* Extracellular enzyme cocktail, *ph-OH* Phenolic hydroxyl

In order to provide more effective evidences for the enzymatic degradation of lignin in Korshinsk peashrub, we established an EEC-based reaction system to verify the direct effect of *D. squalens* on lignin. As shown in Fig. 8a and b, the molecular weight of lignin after the EEC intervention decreased from 6,882.0 to 2,753.0 g/mol, corresponding to a degradation ratio of 40.5%. A significant reduction in phenolic hydroxyl (ph-OH) content was observed in Fig. 8c (*P* < 0.01), which might be attributed to the oxidation of phenolic lignin by laccase and MnP in EEC. FT-IR showed the lignin-related absorption bands at 3,418 cm^−1^, 1,514 cm^−1^, 1,425 cm^−1^, 1,267 cm^−1^ and 1,123 cm^−1^ quickly shifted to lower wavelengths with the EEC intervention (Fig. 8d and additional file 1: Table S12). Nuclear magnetic resonance (NMR) ^13^C spectrum showed that the signal intensity at chemical shifts 55.26 (-OCH_3_), 73.51 (β-O-4), 114.88 (C_5_-H_5_ in G unit) and 128.84 (C_2,6_-H_2,6_ in H unit) quickly weakened with the EEC intervention (Fig. 8e). The presence of ligninolytic enzyme activity, Fe^3+^ reduction capacity, H_2_O_2_ and ·OH in EEC demonstrates that synergistic action of ligninolytic enzymes and free radicals enables continuous lignin degradation (Additional file 2: Fig. S4). These findings confirm that the EEC from *D. squalens* possesses potent lignin-degrading activity.

## Discussion

### Effects of pretreatment with white rot fungi on enzymatic saccharification and in vitro ruminal fermentation of Korshinsk peashrub

The complex cross-linked structure of lignocellulose in woody plants significantly limits its use in ruminant livestock. White rot fungi possess the capability to disrupt these cross-linked structures, then overcoming woody plants recalcitrance. However, current understanding of their roles in enhancing the ruminal degradability of woody biomass remains limited. This study evaluated the biodegradation capacity and patterns of all tested fungi, as well as ruminal degradability of Korshinsk peashrub based on the dynamic changes in the chemical composition and rumen fermentation parameters following fungal treatment. Compared with other fungi, the mycelium growth of *D. squalens* and *Bjerkandera* sp. was fastest during the fermentation (Additional file 2: Fig. S1), indicating the nutrient matrix and fermentation process of Korshinsk peashrub may be more suitable for the *Bjerkandera* sp. and *D. squalens*. Generally, NDF content of Korshinsk peashrub decreased with fungi colonization during the period of 14–28 d. The reduction in NDF was closely related to the degradation of hemicellulose, cellulose and lignin which are a part of NDF. Our results demonstrated that *D. squalens* preferentially degraded lignin, hemicellulose and pectin, consequently cellulose is selectively retained, which caused selective rot in Korshinsk peashrub. Lignin and hemicellulose synchronously decreases under *D. squalens* and *Bjerkandera* sp. treatments, indicating hemicellulose in Korshinsk peashrub is mostly fiber fraction which is bound to lignin. Additionally, we observed a high interaction between fungal species and incubation time, demonstrating that the degradation patterns among fungi vary considerably over time. This phenomenon might be attributed to the fact that fungi have different strategies for synthesizing lignocellulose enzymes at different periods. Among all fungi, the incubation time has the largest impact on *D. squalens*. A prerequisite to trigger synthesis of lignocellulose enzymes from white rot fungi has been confirmed to be starvation of C and N [29]. We observed that activities and expression levels of pectinase, GST, β-glucosidase, esterase, and key lignocellulose enzymes at extended colonization of *D. squalens* have higher values, corresponding to enhanced degradation of lignin, hemicellulose and pectin. Obviously, *D. squalens* began to face a scarcity of C and N during the longer colonization period, potentially driving the synthesis of ligninolytic and carbohydrate enzymes.

Deconstruction of the cross-linked structure of Korshinsk peashrub by biopretreatment has important implications for improving its digestibility in ruminants. Therefore, we evaluated the digestion and utilization of Korshinsk peashrub for ruminants by in vitro rumen fermentation and gas system before feeding. It has been documented that incorporating *Pleurotus eryngii* into corn straw for 28 d led to a lignin degradation rate of 25.0% and a net increase of 7.5% in IVDMD, with minimal cellulose loss [30]. Similarly, Niu et al. [31] demonstrated that pretreatment of wheat straw with the white rot fungus *Irpex lacteus* resulted in a lignin degradation rate of 28.0%, which correspondingly improved the rumen and enzymatic digestibility by 18.0% and 34.0%*.* These findings are consistent with our results, showing that pretreatment with *D. squalens* caused a net reduction of 27.4% in lignin, which was accompanied by a 7.1% increase in IVDMD and a 48.9% improvement in enzymatic digestibility. Recently, Ge et al. [32] confirmed that the increases in IVDMD and enzymatic digestibility are primarily attributed to the preferential degradation of lignin and hemicellulose by *P. ostreatus *and* I. lacteus*. Therefore, the removal of lignin and its linkages with carbohydrates in our study may be a pivotal factor affecting rumen digestibility. However, this interpretation requires further verification.

Cellulose is a key nutrient available to ruminal microorganisms. Therefore, cellulose bioaccessibility in Korshinsk peashrub was evaluated by in vitro RSY using commercial cellulase. After 28 d of incubation with *D. squalens*, the degradation of hemicellulose, lignin, and pectin reached the highest levels, significantly exceeding those observed in all other fungi. Three-dimensional structure of lignin and its linkages with hemicellulose form a natural recalcitrance barrier that embeds cellulose, thereby hindering enzymatic hydrolysis and microbial adhesion [33]. Additionally, cellulose saccharification is further impeded by strong intermolecular hydrogen bonding and high crystallinity [34]. In our study, the enzymatic hydrolysis efficiency increased with the time, indicating enhanced interaction between cellulase and its substrate. This phenomenon might result from white rot fungi removing the lignin barrier through the oxidative action of hydroxyl radicals and MnP, thereby improving cellulose accessibility and subsequent enzymatic saccharification [35]. Currently, there are limited reports on the influence of pectin content and its spatial distribution in biomass on enzymatic saccharification. Pectin can serve as a determining factor for the porosity and rigidity of the cell wall due to its hydrophilic and cross-linking properties [36]. During *D. squalens* colonization, the pectin content in Korshinsk peashrub decreased significantly over time. It has been documented that pectin removal increases porosity and surface area, as observed in *Cannabis sativa* (hemp), leading to a 26.0% increase in cellulose hydrolysis rate [37]. This finding is consistent with our results. Mounting evidence suggests that pectin is tightly anchored to the surface of cellulose in the plant cell wall through extensive physical contacts within 1 nm, forming a rigid structure rather than loose structures [38]. If this is true, the removal of pectin will expose crystalline cellulose and thus increase cellulose bioaccessibility. Overall, the removal of lignin, hemicellulose, and pectin by *D. squalens* may be closely related to ruminal cellulose degradation.

The soluble fraction [39] and lignin-derived aromatic compounds [40] in the matrix may determine ruminal IVGP. A net increase of NDS in *D. squalens* treatment would provide a directly available carbon source for rumen microbes, which may therefore be the reason for improved IVGP. Furthermore, delignification by *D. squalens* and *Bjerkandera* sp. can enhance access of rumen microbes and extracellular enzymes to the fibers, thereby promoting the cellulose digestion in ruminants. It has been reported that lignin degradation products, such as vanillin, vanillic acid, and *p*-hydroxybenzoic acid, syringic acid and cinnamic acid, can inhibit rumen microorganisms growth by disrupting membrane integrity, thereby delaying rumen fermentation [40]. Worth noting is that lignin degradation by *D. squalens* and *Bjerkandera* sp. may cause a net increase of lignin-derived aromatic compounds. However, ruminal IVGP significantly increased. Growing evidence suggests that these aromatic compounds can be transported into the cell via membrane transporters, where oxidative decarboxylases or hydroxylases will introduce hydroxyl groups to create ring‑opening sites. Subsequently, these hydroxylated aromatic compounds are further cleaved by dioxygenases and then converted into amino acids and lipids in intracellular central carbon metabolism [41, 42]. Thus, unique intracellular metabolic pathways of white rot fungi may reduce the concentration of aromatic inhibitors in the matrix, thereby mitigating their toxicity to rumen microbes. Considering that *D. squalens* not only led to a net increase in NDS coupled with a reduction in lignin, hemicellulose and pectin, but also resulted in higher IVDMD and IVGP, it has significant potential in enhancing the rumen degradation of Korshinsk peashrub.

Rumen fermentation products reflect dietary carbohydrate digestion. TVFAs concentration significantly increased in *D. squalens* treatment compared with the CON, directly reflecting the increase in ruminal Korshinsk peashrub digestibility. Acetate and propionate, which are rapidly absorbed by the rumen papillae, serve as crucial substrates for lipid and glucose synthesis in ruminants [26]. *D. squalens* treatment significantly decreased acetate-to-propionate ratio with enhanced propionate, indicating a shift toward a propionate-dominant fermentation pattern that may enhance energy supply to the ruminants. The selective characteristic of *D. squalens* can cause a significant degradation of hemicellulose, pectin and lignin with slight cellulose loss, directly resulting in the accumulation of soluble carbohydrates. The increase in dietary soluble carbohydrates has been reported to enhance propionate production by activating propionate-type bacteria in the rumen [43]. After *D. squalens* intervention, the NH_3_-N, isobutyrate, and isovalerate concentrations were significantly reduced. This observed effect is likely due to the synergistic actions of multiple genes in fungi, including nitrogen transport protein, glutamine synthetase, fructose-6-phosphate aminotransferase and chitin synthase, will introduce nitrogen from the nutrient matrix into the chitin biosynthetic pathway [44, 45]. Chitin, a recalcitrant component of fungi cell wall, effectively prevented rumen microbes from fermenting dietary protein into branched-chain fatty acids.

### Lignin decomposition system of *D. squalens*

The phenolic units under laccase action were oxidized into phenoxyl radicals, which then participated in either radical rearrangement or coupling-based polymerization, followed by C_*α*_-C_*β*_ cleavage in side chain and C_*α*_ oxidation, and alkyl-aryl cleavage of aromatic rings in lignin [46]. Additionally, laccase, as an electron acceptor, generates stable radical intermediates by oxidizing natural mediators, such as 3-hydroxyanthranilic acid, violuric acid, and ABTS [47]. These intermediates then diffuse into the intricate structure of lignin until the cleavage of β-O-4, β-1, β-β linkages in non-phenolic lignins. MnP oxidizes Mn^2+^ to Mn^3+^ with the aid of H_2_O_2_ and then attacks phenolic-type lignin. MnP has also been proven to cleave non-phenolic lignin via small mediators in matrix, such as unsaturated lipid or thiyl radicals [48]. Versatile peroxidase, known as its broad substrate specificity, is capable of oxidizing Mn^2+^, phenolic lignin, and non-phenolic lignin. Several earlier studies had demonstrated that MnP and GST disrupt β-O-4 aryl ether bonds in lignin [35, 49], thereby depolymerizing lignin into aromatic units. Worth noting is that the carbohydrate esterases, such as 4-O-methyl-glucuronoyl methylesterases found in extracellular secretomes, contribute to the uniform deletion of lignin and hemicellulose by cleaving the ester linkages connecting glucuronoxylan to lignin [12]. Obviously, the presence of esterase will inevitably accelerate the removal of the lignin–hemicellulose barrier without significantly compromising lignocellulose matrix. However, cellulose degradation does not benefit from this mechanism, which may explain why *D. squalens* preferentially degrades lignin and hemicellulose. Furthermore, our secretome analysis revealed that hemicellulases exhibited both higher activity and expression levels than cellulases, thereby accelerating hemicellulose degradation. Due to its low relative abundance, the oxidative decomposition of lignin by dye-decolorizing peroxidase is limited. Therefore, we inferred that the removal of lignin primarily benefited from the laccase, MnP, versatile peroxidase, GST, and esterase.

Lignin auxiliary enzymes secreted by *D. squalens* play a significant role in the decomposition process of Korshinsk peashrub. Our findings displayed that Fe^3+^ reduction and H_2_O_2_ production capacities were significantly enhanced after fungal intervention, accompanied by the concurrent generation of hydroxyl radicals, indicating that the Fenton reaction was effectively triggered. During decomposition, CDH activity increased significantly over time. The CDH consists of two domains, one is a dehydrogenase domain containing flavin adenine dinucleotide (FAD), and the other is a cytochrome domain containing *b*-type heme. In addition to its function in H_2_O_2_ production, CDH as the electron donor can reduce Fe^3+^ to Fe^2+^ through its iron-reducing domain [50]. After the dehydrogenase domain of CDH oxidizes the anomeric C_1_ position of cellobiose, electrons are sequentially transferred from the reduced FAD in dehydrogenase domain to heme b in the cytochrome domain, and ultimately to an external electron acceptor such as Fe^3+^ [51]. These results preliminarily confirmed that CDH may drive the Fenton reaction to produce hydroxyl radicals by reducing Fe^3+^ and generating H_2_O_2_, contributing to lignin deconstruction.

### Structural changes of Korshinsk peashrub deconstruction by *D. squalens*

The deconstruction of lignin and carbohydrates by the enzymatic and free radical system is inevitably accompanied by changes in the surface structure and chemical bonds of the Korshinsk peashrub. Cellulose is the sole constituent of the plant cell wall that contains crystalline regions [34]. Therefore, we attribute increased crystallinity of Korshinsk peashrub to the preferential degradation of *D. squalens* on its amorphous components, such as lignin, hemicellulose and pectin. There were clear declines at the bands 1,319 cm^−1^ and 1,242 cm^−1^, corresponding to the phenoxethyl ether C-OCH_3_ vibration of syringyl and guaiacyl units in lignin, respectively [52]. This suggests that *D. squalens* attacked side chains in lignin. The absorption band at 1,242 cm^−1^ is also defined as the ester bond between hemicellulose and lignin [53]. The bands at 1,510 cm^−1^, 1,425 cm^−1^ and 1107 cm^−1^ are assigned to C-C vibration or C-H deformation of the aromatic ring in lignin [54]. The absorption peaks quickly declined after the fungal pretreatment, revealing *D. squalens* can cause the cleavage of lignin skeleton structure. The absorption bands at 3,420 cm^−1^ may be ascribed to stretching vibration of aromatic -OH group [54], and this band was quickly weakened after *D. squalens* intervention. This suggested *D. squalens* could cause the oxidative destruction of the phenolic structure in lignin. The unique ability of laccase and MnP is to specifically attack phenolic lignin. There were clear declines at the bands 1,736 cm^−1^ and 1,628 cm^−1^, corresponding to the acetyl group or carboxyl group in hemicellulose and pectin [55]. The absorption bands at 1,053 cm^−1^ and 897 cm^−1^ correspond to the C-O stretch or C-H deformation of cellulose, hemicellulose and pectin [56], and significantly declined after the fungal pretreatment. This showed that *D. squalens* led to a uniform bioconversion of cellulose, hemicellulose and pectin in Korshinsk peashrub. Under the ECC action, the molecular weight of lignin significantly decreases with a degradation ratio of 40.5%. ^13^C NMR spectrum showed that the -OCH_3_, β-O-4, C_5_-H_5_ in G unit and C_2,6_-H_2,6_ in H unit of lignin are quickly broken after the EEC intervention. These results further support that *D. squalens* drives the lignin degradation in Korshinsk peashrub.

Following *D. squalens* treatment, the surface of Korshinsk peashrub exhibited a notable emergence of macropores, characterized by an increased average pore size and specific surface area. The N_2_ adsorption and desorption curves are characterized by typical type-II and type-IV profiles according to the International Union of Pure and Applied Chemistry classification [57], indicating the emergence of abundant mesopores or macropores in Korshinsk peashrub with *D. squalens* intervention. This change is likely due to the degradation of lignin, hemicellulose, and pectin in Korshinsk peashrub. The dye DO15 (5–36 nm) can simulate the bioaccessibility of cellulase to substrates, because it has a similar size to cellulase [27]. A significant increase in the adsorption capacity of DO15 and cellulase in Korshinsk peashrub indicates that *D. squalens* can enhance the cellulose bioaccessibility.

Several limitations of this study should be considered when interpreting the results. Only four fungal species were evaluated under controlled laboratory conditions, which may not represent all relevant white rot fungi in natural or industrial settings; other fungi or different fermentation conditions might yield distinct degradation patterns and efficiency. IVDMD, IVGP and rumen fermentation products were evaluated based on in vitro rumen fermentation system, which did not fully simulate the complexity of the in vivo ruminal environment and long‑term feeding outcomes. Future studies will include in vivo trials on ruminants to validate the biopretreatment effects of *D. squalens* observed in this study.

## Conclusions

Herein, we evaluated the biodegradability, enzymatic saccharification and in vitro rumen fermentation of Korshinsk peashrub after pretreatment with four fungi. Among all fungi, *D. squalens* is the most effective degrader of Korshinsk peashrub, and its genome possesses a comprehensive range of genes encoding ligninolytic enzymes. *D. squalens* preferentially degraded lignin, hemicellulose, and pectin via its free radicals system and lignocellulolytic enzymes, consequently cellulose was selectively retained, which caused a selective rot in Korshinsk peashrub. Selective rot of *D. squalens* resulted in the cleavage of the resistant cross-linked structures in Korshinsk peashrub, thereby enhancing both enzymatic saccharification and in vitro rumen fermentation. In conclusion, the biopretreatment process using *D. squalens* or its lignocellulolytic enzymes upgrades Korshinsk peashrub into high‑quality ruminant feed.

## Supplementary Information

Additional file 1: Table S1. Composition and nutrient levels of basal diets. Table S2. Chemical compositions analysis of Korshinsk peashrub decayed by different white rot fungi. Table S3. Chromosomal localization of predicted genes in D. squalens. Table S4. Statistics of genomic contigs and scaffolds of D. squalens after patching the bases gaps. Table S5. Total data size and sequencing coverage of the genome of D. squalens. Table S6. Summary of KEGG, KOG and GO annotation of genes from D. squalens. Table S7. Top 70 Pfam domains in D. squalens genome. Table S8. CAZy annotation of genes from D. squalens. Table S9. Comparative analysis of ligninolytic enzymes, cellulase, hemicellulase and pectinase of 11 sequenced white rot fungi. Table S10. Genes and their translation levels related to the degradation of plant cell wall polysaccharides under different incubation periods in D. squalens. Table S11. Peaks attribution and signal intensities in FT-IR spectra of Korshinsk peashrub after D. squalens pretreatment. Table S12. Peaks attribution and signal intensities in FT-IR spectra of lignin.

Additional file 2: Fig. S1 Growth status of different white rot fungi during fermentation. Fig. S2 Functional annotation of extracellular enzymes secreted by D. squalens. Fig. S3 N2 adsorption-desorption isotherms and pore size distribution curves of Korshinsk peashrub following D. squalens pretreatment. Fig. S4 Ligninolytic catalytic network of the extracellular enzyme cocktail.

## Data Availability

Correspondent authors upon reasonable requests will provide raw data supporting the results of this study.

## Abbreviations

AA: Auxiliary activities
ABTS: 2,2-Azino-bis-3-ethylthiazoline-6-sulfonate
CAZy: Carbohydrate-active enzymes
CDH: Cellobiose dehydrogenase
DB1: Direct blue 1
DNS: 3,5-Dinitrosalicylic acid
DO15: Direct orange 15
DW: Dry weight
EEC: Extracellular enzyme cocktail
FAD: Flavin adenine dinucleotide
FT-IR: Fourier transform infrared spectroscopy
GO: Gene Ontology
GST: Glutathione *S*-transferase
IVDMD: In vitro dry matter digestibility
IVGP: In vitro gas production
KEGG: Kyoto Encyclopedia of Genes and Genomes
KOG: Eukaryotic Orthologous Groups
MnP: Manganese peroxidase
NDF: Neutral detergent fiber
NDS: Neutral detergent solubles
NMR: Nuclear magnetic resonance
OM: Organic matter
Pfam: Protein family
ph-OH: Phenolic hydroxyl
RSY: Reducing sugar yield
SEM: Standard error of the mean
TBARS: Thiobarbituric acid reactive substances
TVFAs: Total volatile fatty acids
WSC: Water soluble carbohydrates
XRD: X-ray diffraction
