# Supplementary material for: Enhanced valorization of Korshinsk peashrub (Caragana korshinskii Kom.) as ruminant feed via the selective ligninolysis by the white rot fungus Dichomitus squalens
Source: J Anim Sci Biotechnol. 2026 May 12;17:90. doi: 10.1186/s40104-026-01404-z (PMC13162467; doi:10.1186/s40104-026-01404-z)
Supplement: Supplementary file 2 — Additional file 2: Fig. S1 Growth status of different white rot fungi during fermentation. Fig. S2 Functional annotation of extracellular enzymes secreted by D. squalens. Fig. S3 N2 adsorption-desorption isotherms and pore size distribution curves of Korshinsk peashrub following D. squalens pretreatment. Fig. S4 Ligninolytic catalytic network of the extracellular enzyme cocktail. [file 40104_2026_1404_MOESM2_ESM.docx]

**Supplementary Methods**

**Assembly and gene structure prediction of Telomere-to-Telomere (T2T) genome of *Dichomitus squalens***

**Assembly**

Fungal genomic DNA was extracted using the EasyPure® Genomic DNA Kit (EE101; TransGen Biotech, Beijing, China). To construct contigs, three distinct methodologies were rigorously applied. Initially, Nanopore ultra-long sequencing data were assembled using Nextdenovo v2.5.2 [1]. Subsequently, PacBio HiFi data were assembled with hifiasm v0.19 [2]. In a final integrative approach, both Nanopore ultra-long and PacBio HiFi datasets were combined using hifiasm v0.19. After thorough comparison of the assemblies derived from these strategies, the assembly generated using hifiasm v0.19 alone was deemed superior for subsequent analyses.

For Hi-C reads, quality control was meticulously performed using fastp v0.23.4 (https://github.com/OpenGene/fastp, a widely-recognized tool for preprocessing sequencing data. Alignment was then executed with bowtie2 v2.3.2, employing stringent parameters: -end-to-end, -very-sensitive, and -L 30, to ensure high-fidelity mapping. Subsequently, contigs were subjected to clustering, ordering, and orientation using LACHESIS [3], a sophisticated tool for scaffolding genome assemblies.

The resulting draft genome, comprising 13 chromosomes, was further refined. Nanopore ultra-long data were strategically applied to bridge sequence gaps and to assemble telomeric regions, thereby achieving a gapless draft genome assembly. To rectify any potential errors in the genome sequences, NextPolish2 was employed, leveraging both WGS and PacBio HiFi reads for comprehensive correction [4].

**Gene structure prediction**

In order to improve the accuracy of prediction results, multiple gene prediction tools were used for comprehensive analysis [5]. Firstly, the Augustus software was used to predict genes de novo based on the gene structure characteristics of the sequenced congeneric species (https://www.ncbi.nlm.nih.gov/datasets/genome/GCF_000275845.1/), and identified all structural genes in the genome sequence. Taking the protein sequences of closely related species as references, Genewise prediction was conducted. Then, the results obtained by the two methods were integrated using EVidenceModeler 2.0 (https://github.com/EVidenceModeler/EVidenceModeler/releases) and verified in the second round of Program to Assemble Spliced Alignments software (https://github.com/PASApipeline/PASApipeline) to obtain the *D. squalens* structural gene set.

**Genes function annotation**

All genes were annotated to the Gene Ontology (GO) database (http://geneontology.org/) for functional classification. Enrichment analysis of genes was conducted using the online Kyoto Encyclopedia of Genes and Genomes (KEGG) database (http://www.genome.jp/kegg/) to identify relevant metabolic pathways. Protein sequence of all genes can be annotated to a specific Cluster of Orthologous Groups (COG; http://www.ncbi.nlm.nih.gov/COG/) and each COG cluster is composed of orthologous sequences. Furthermore, all protein sequences were annotated into the pfam (http://pfam.xfam.org/) database for gene annotation, protein family classification, and prediction of gene function and structure

**Phylogenomic analysis of *D. squalens* and 10 typical WRF species based on gene families**

Phylogenomic analysis based on gene families was performed following a previously established approach [5]. CDS sequences, genome assembly sequences, gff and PEP files of 11 white-rot fungi (WRF) that have undergone whole-genome sequencing were downloaded from the classification of Polyporales in the basitiomycetes class of the JGI (Joint Genome Institute) database (https://mycocosm.jgi.doe.gov/mycocosm/home). Solar software was used to remove redundant sequences, and then the gene family clustering results was obtained by Hcluster-sg software. Based on the results of single-copy direct homologous genes, MUSCLE 5.0 software (https://www.drive5.com/muscle5/) and MAFFT 7.0 (https://mafft.cbrc.jp/alignment/software/) was used for protein and CDS multi-sequence alignment, and finally an evolutionary tree was constructed based on the maximum likelihood model of Treebest. The 11 WRF included *D. squalens* (Sequencing completed in our study), *Irpex lacteus* (IL), *Phanerochaete chrysosporium* (PC), *Ceriporiopsis subvermispora* (CS), *Trametes versicolor* (TV), *Bjerkandera adusta* (BA), *Pleurotus eryngii* (PE), *Lentinula edodes* (LE), *Pleurotus ostreatus* (PO), *Phlebia radiata* (PR) and *Ganoderma leucocontextum* (GL).

**Extracellular proteomic analysis of *D. squalens***

**Sample preparation and enzyme protein separation**

The fungal cultures at the different periods were mixed with sterile water at a ratio of 1:4 (g/mL), extracted for 2 h at 4℃, 200 rpm, and then centrifuged for 20 min at 4℃, 12000 × *g*. The filtrates (crude enzyme solution) were subjected to subsequent tests. The crude enzyme solution was taken out of a -80℃ refrigerator and thawed at 4℃. Then, it was centrifuged at 12,000 × *g* at 4℃ for 20 min to obtain the supernatant. After vacuum freeze-drying, 50 mL of pre-cooled trichloroacetic acid and acetone mixture was added and precipitated at -20 ℃ for 6 h. Centrifugation was performed at 12,000 × *g* for 30 min at 4℃, the supernatant was discarded, and the protein precipitate was retained. The procedure was repeated 2 to 4 times, all proteins were merged, and freeze-dried under vacuum.

**Tryptic digestion**

Protein was digested by FASP (Filter-Aided Sample Preparation) method. To perform trypsin digestion, the protein pellets were first resuspended in a solution containing 8 M urea (Solarbio, Beijing, China) and 100 mM NH_4_HCO_3_ (Solarbio, Beijing, China). The concentration of the proteins was then determined using the BCA protein assay, and a quantity of 1 mg of protein was carefully isolated for further processing. The reduction step was initiated by adding 500 mM dithiothreitol (DTT; Thermo Scientific, Rockford, IL) to achieve a final concentration of 5 mM within the samples. These were then incubated at 60°C for 30 minutes and shaken continuously at 1000 rpm to ensure thorough mixing and reduce disulfide bonds. To decrease the salt concentration, the samples were diluted tenfold with 100 mM ammonium bicarbonate. Additionally, 1 M CaCI_2_ (Solarbio, Beijing, China) was incorporated into the mixture to attain a final sample concentration of 1 mM CaCl₂, which is essential for the subsequent digestion process. Trypsin (USB Trypsin, Promega, Madison, WI) was introduced at a ratio of 1 µg of trypsin per 50 µg of protein. The digestion was carried out at 37°C for 3 hours, with the samples being shaken at 850 rpm to maintain optimal conditions for enzymatic activity. Following digestion, the samples underwent desalting using 1 mL Discovery C18 SPE columns (Supelco, Bellefonte, PA) to remove any residual salts and impurities. The desalted samples were then concentrated to a volume of 100 µL using a speed vac. Finally, the concentration of the resulting peptides was measured using the BCA protein assay. The peptides were aliquoted into vials at a concentration of 0.1 µg/µL, with a total volume of 50 µL, and prepared for mass spectrometry (MS) analysis.

**Liquid** **chromatography with tandem mass spectrometry (LC-MS/MS) analysis**

Peptide separation was performed using a Vanquish Neo UHPLC system (Thermo Scientific) equipped with a Trap Column (5 µm C18, 300 µm × 5 mm, PepMap Neo, Thermo Scientific). The mobile phases consisted of 0.1% formic acid in water (Phase A) and 0.1% formic acid in 80% acetonitrile (Phase B). The gradient elution conditions were as follows: 0-0.1 min, B solution gradient 4%-6%; 0.1-1.1 minutes, B solution gradient is 6%-12%; 1.1-4.3 min, B solution gradient is 12%-22.5%; 4.3-6.1 min, B solution gradient is 22.5%-45%; 6.1-8 min, the B solution was maintained at 99%. The separated peptides were analyzed by an Orbitrap Astral mass spectrometer (Thermo Scientific) using data-independent acquisition (DIA) mode. The mass spectrometry conditions were set as follows: precursor ion scan range, 380–980 m/z; total time, 8 min; ion mode, positive; electrospray voltage, 2.2 kV; resolution for MS1, 240,000; maximum ion time for MS1, 3 ms; AGC (automatic gain control) target, 500%; resolution for MS2, 80,000; maximum ion time for MS2, 3 ms; AGC target, 500%; RF-lens relative voltage, 40%; MS2 activation type, collision dissociation; isolation window, 2 Th; cycle time, 0.6 s; normalized collision energy, 25%.

**Lignin degradability of extracellular enzyme cocktail (EEC)**

**Reaction system of lignin degradation by EEC**

To verify the degradation effects of EEC from *D. squalens* on lignin, we constructed a reaction system of EEC and lignin. After 28 d of incubation, the solid-state fermentation cultures of Korshinsk peashrub by *D. squalens* were thoroughly mixed with sterile water at a ratio of 1:4 (g/mL), extracted for 2 h at 4℃, 200 rpm. The filtrates were collected after centrifugation at 4℃ and 12,000 × *g* for 20 minutes. The filtrates was pre-frozen in a -80℃ refrigerator for 8 h, followed by vacuum freeze-drying using a vacuum freeze dryer (CHRIST, Germany). Enzymatic hydrolysis was performed in a 6 mL of reaction system containing 1.0 g dry EEC and 0.1 g lignin substrate. After enzymatic hydrolysis for 72 h at 50℃, 800 rpm, the hydrolyzed substrates were centrifugated at 12,000×*g*, 4°C for 30 min and the lignin precipitate was collected. The lignin after enzymatic hydrolysis was washed 2-3 times with ultrapure water and dried at 55℃ to constant weight for subsequent analysis.

**Analysis of degradation ratio, phenolic hydroxyl (ph-OH) content and molecular weight of lignin**

After the enzymatic hydrolysis reaction for 72 h, 100 μL of the reaction solution was added to a volumetric flask containing 10 mL of 1, 4-dioxane, shaken at 600 rpm for 5 min, and the total volume was constant to 50 mL. The absorbances of the diluted reaction solution at 280 nm were measured before and after enzymatic hydrolysis. The lignin concentration was calculated through the standard curve of lignin (*y* = 0.0146*x*-0.0122, R^2^ = 0.9929) and absorbance values. The degradation ratio of lignin degradation was calculated based on the differences in lignin concentration before and after enzymatic hydrolysis.

The molecular weight of alkali lignin was determined by Differential Refractive Index Detector using Gel Permeation Chromatography (Agilent 1260 Infinity, USA) equipped with a Shodex KD-802 column. The mobile phase is N,N-dimethylformamide and the standard sample is Polymethyl methacrylate. The detection conditions are mainly as follows: column temperature, 40℃; flow rate, 1 mL/min.

The phenolic hydroxyl groups (ph-OH) in lignin (DQ-2, Shengquan Group Share-Holding Co., Ltd., Shandong, China) were quantified based on an ionization difference UV–Vis method [6-7].

The functional groups of lignin before and after pretreatment were analyzed using a Bruker AVANCE III HD 600 MHz solid-state nuclear magnetic resonance ¹³C spectrometer equipped with a 5 mm high-resolution spinning probe (Bruker, Switzerland). The specific parameter settings are as follows: pulse sequence, zg30; number of scans, 16; relaxation delay, 1.0 s; pulse width, 10.91 μs; acquisition time, 5 s.

**Supplemental figures**


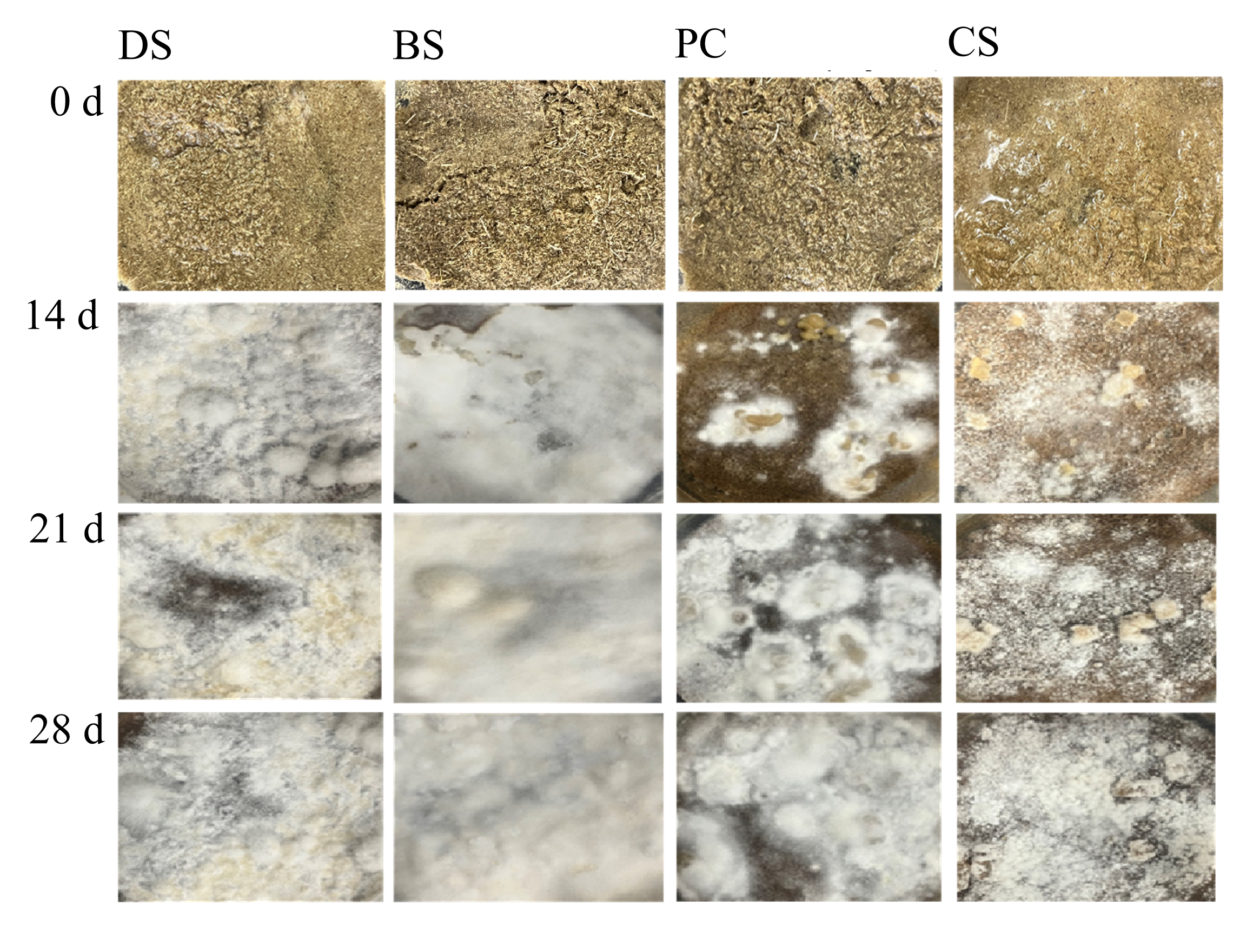


**Fig. S1** Growth status of different white rot fungi during fermentation. *DS* *Dichomitus squalens,* *BS* *Bjerkandera* sp.*,* *PC* *Phanerodontia chrysosporium,* CS *Ceriporiopsis subvermispora*


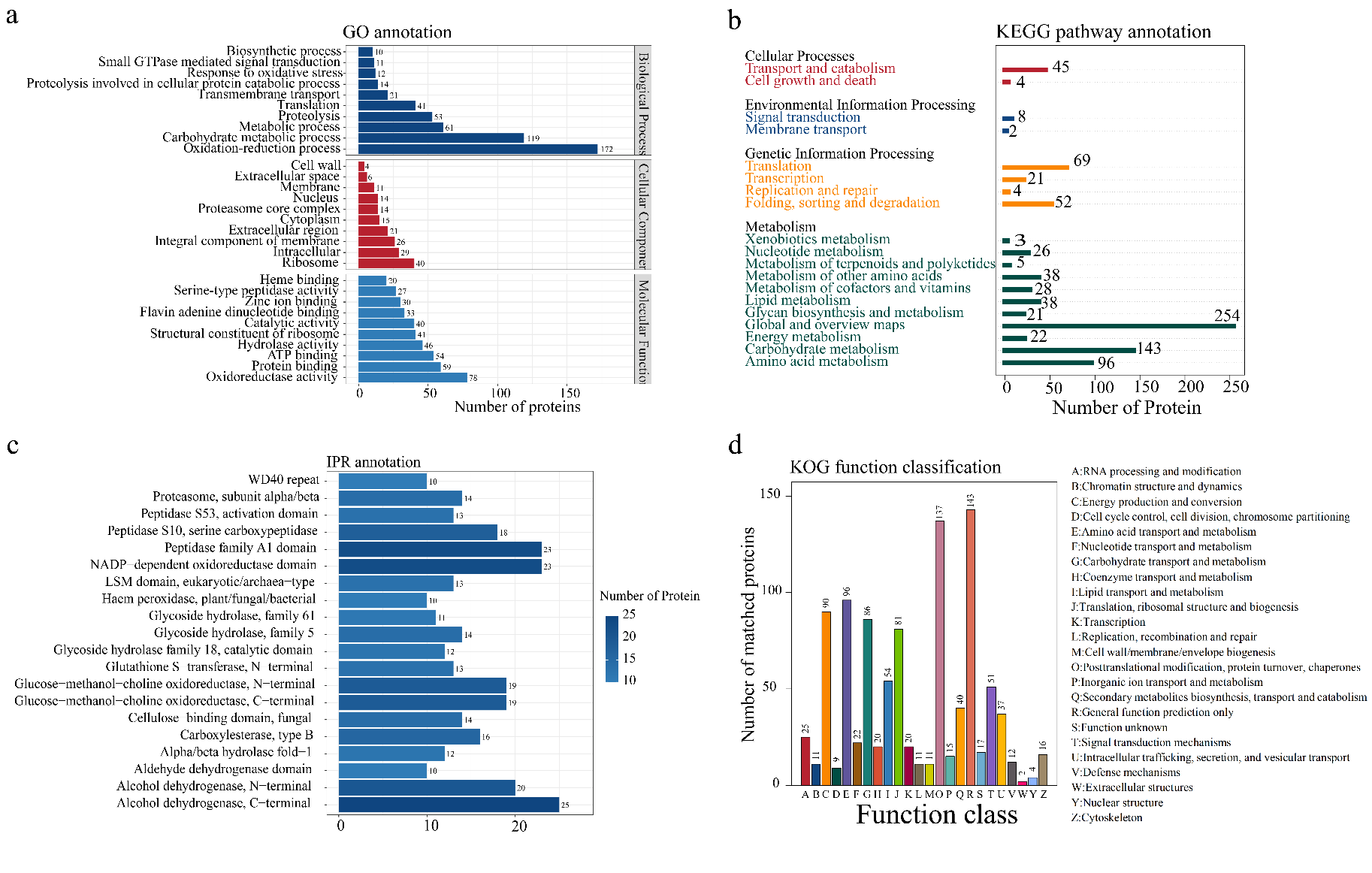


**Fig. S2** Functional annotation of extracellular enzymes secreted by *D. squalens*. **a** GO annotation. **b** KEGG annotation. **c** IPR annotation. **d** KOG annotation. *KEGG* Kyoto Encyclopedia of Genes and Genomes, *GO* Gene Ontology, *IPR* InterPro, *KOG* EuKaryotic Orthologous Groups


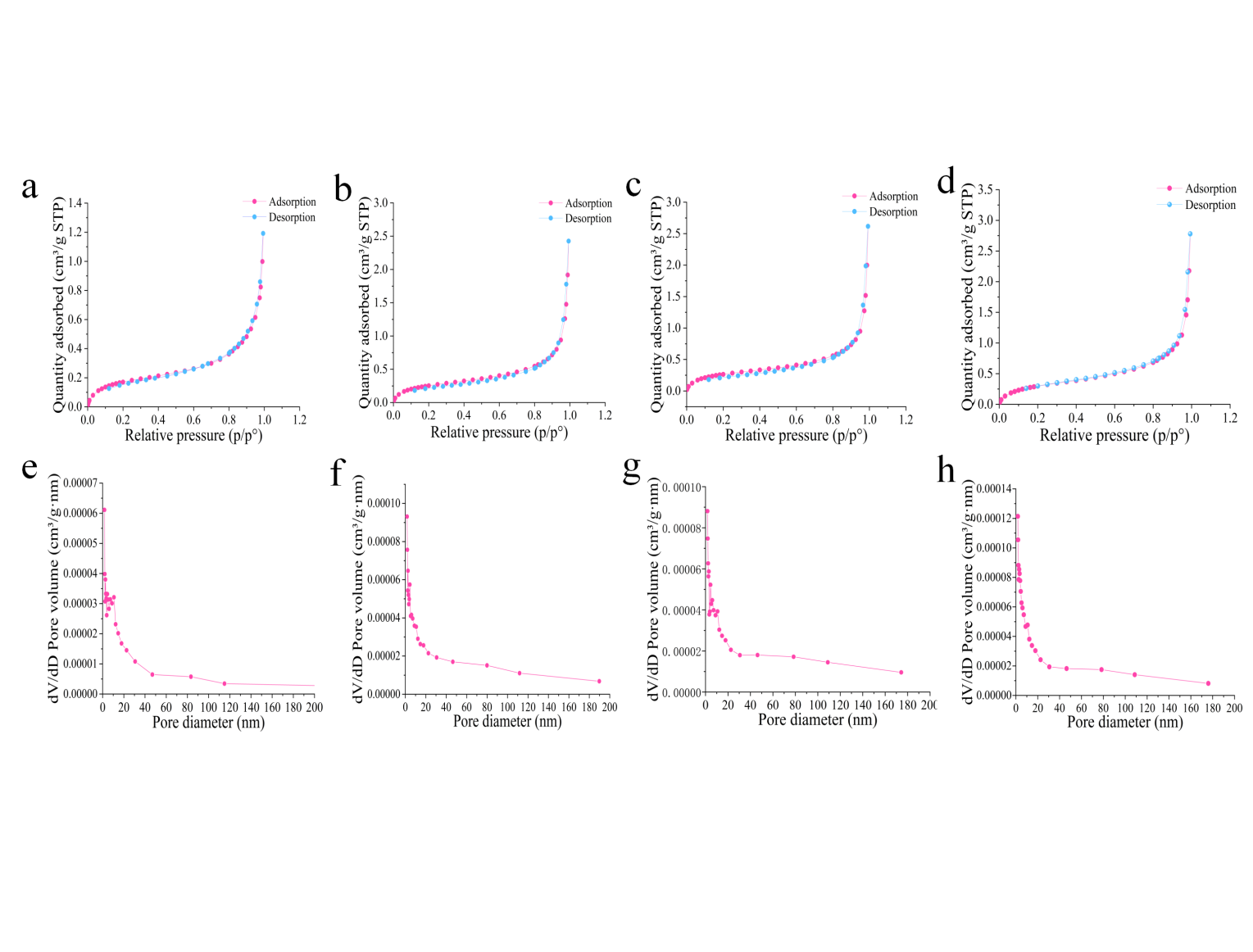


**Fig. S3** N_2_ adsorption-desorption isotherms and pore size distribution curves of Korshinsk peashrub following *D. squalens* pretreatment. **a**–**d** The N_2_ adsorption and desorption curves after *D. squalens* submerged culture for 0, 14, 21, and 28 d. **e**–**h** The pore size distribution curves after *D. squalens* submerged culture for 0, 14, 21, and 28 d. *P/P^o^* Relative pressure

**
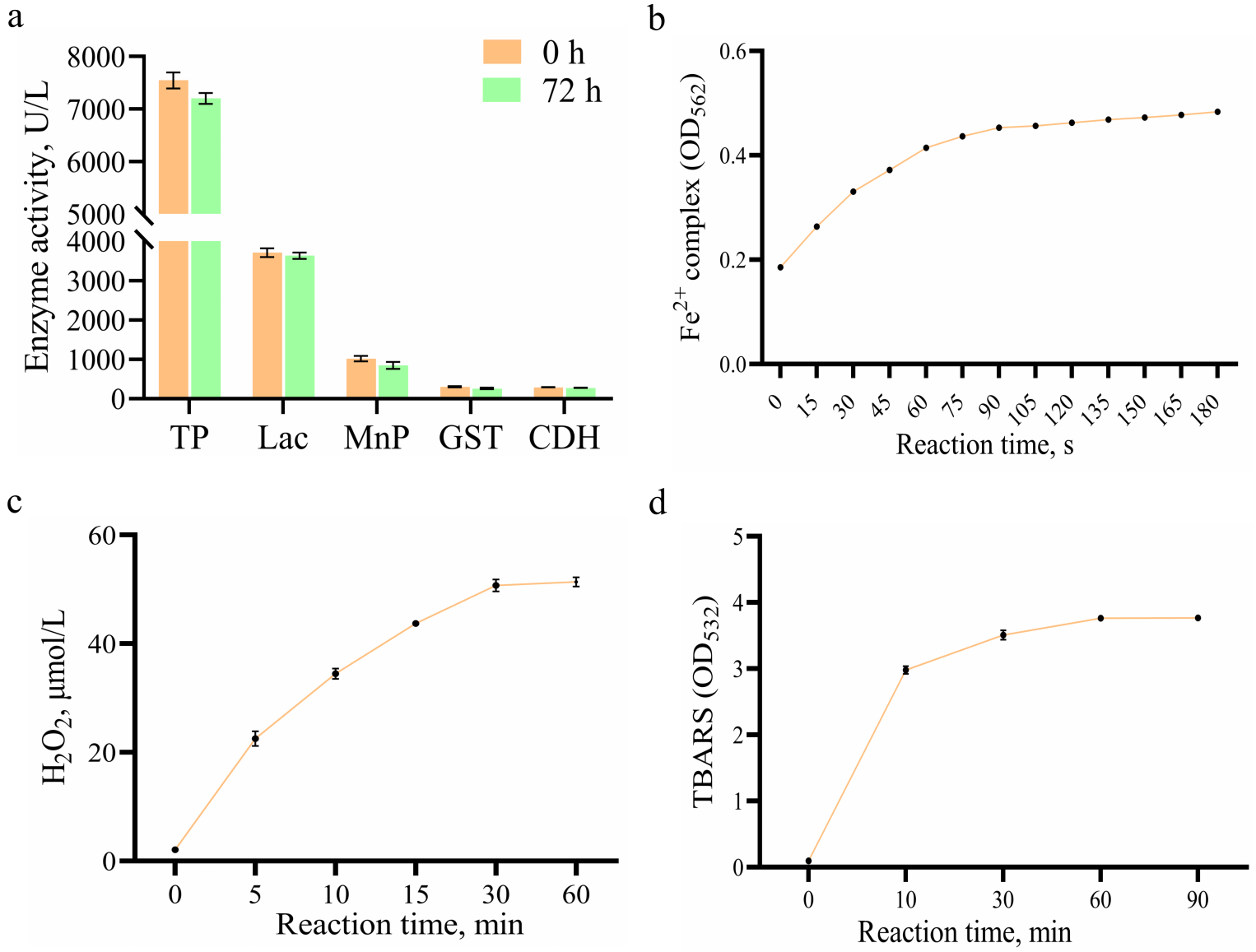
**

**Fig. S4** Ligninolytic catalytic network of the extracellular enzyme cocktail. **a** Activity of lignin-related enzymes, including TP, Lac, MnP, GST and CDH; **b** Fe^3+^ reduction capacity; **c** H_2_O_2_ production capacity; **d** ^•^OH production capacity. *TP* Total peroxidase, *Lac* Laccase, *MnP* Manganese peroxidase, *GST* Glutathione-S-transferase, *CDH* Cellobiose dehydrogenase

**References**

1. Hu J, Wang Z, Sun Z, Hu B, Ayoola AO, Liang F, et al. NextDenovo: an efficient error correction and accurate assembly tool for noisy long reads. Genome Biol. 2024b;25:107.
2. Cheng H, Concepcion GT, Feng X, Zhang H, Li H. Haplotype-resolved de novo assembly using phased assembly graphs with hifiasm. Nat Methods. 2021;18:170-175.
3. Burton JN, Adey A, Patwardhan RP, Qiu R, Kitzman JO, Shendure J, et al. Chromosome-scale scaffolding of de novo genome assemblies based on chromatin interactions. Nat Biotech. 2013;31:1119-1125.
4. Hu J, Wang Z, Liang F, Liu S, Ye K, Wang D. NextPolish2: A repeat-aware polishing tool for genomes assembled using HiFi long reads. Genomics Proteomics Bioinf. 2024;22:qzad009.
5. Qin X, Su XY, Luo HY, Ma R, Yao B, Ma FY. Deciphering lignocellulose deconstruction by the white rot fungus *Irpex lacteus* based on genomic and transcriptomic analyses. Biotechnol Biofuels. 2018;11:58.
6. Chen L, Wei XX, Wang H, Yao M, Zhang LM, Gellerstedt G, et al. A modified ionization difference UV–vis method for fast quantitation of guaiacyl-type phenolic hydroxyl groups in lignin. Int J Biol Macromol. 2022;201:330-337.
7. Chen JZ, Lu F, Si XQ, Nie X, Chen JS, Lu R, et al. High Yield Production of Natural Phenolic Alcohols from Woody Biomass Using a Nickel-Based Catalyst. Chemsuschem. 2016;9(23):3353-3360.
